# Effects of poultry by-product meal and complete replacement of fish oil with alternative oils on growth performance and gut health of rainbow trout (*Oncorhynchus mykiss*): a FEEDNETICS™ validation study

**DOI:** 10.1186/s12917-024-04324-0

**Published:** 2024-10-17

**Authors:** Imam Hasan, Simona Rimoldi, Biagina Chiofalo, Marianna Oteri, Micaela Antonini, Rosangela Armone, Violeta Kalemi, Laura Gasco, Genciana Terova

**Affiliations:** 1 Department of Biotechnology and Life Sciences, University of Insubria, Varese, 21100 Italy; 2 Department of Veterinary Sciences, University of Messina, Messina, Italy; 3 Department of Agricultural, Forest and Food Sciences, University of Turin, Grugliasco, TO Italy

**Keywords:** Alternative protein sources, Fishmeal substitution, Poultry by-product meal, Growth performance, Rainbow Trout, Sustainable aquafeed, FEEDNETICS™

## Abstract

**Background:**

Aquaculture, traditionally a form of biotechnology, has evolved to integrate innovative biotechnological applications, such as advanced feed formulations, aimed at improving the growth performance and health of farmed fish species. In the present study, the effects of feeding rainbow trout with novel feed formulations were investigated. Fish growth, gut and liver morphology, the concentration of fatty acids in the fillet, and volatile fatty acids in the gut were assessed. The study also validated scenarios from in vivo experiments using a nutrient-based model called FEEDNETICS™. This globally used model serves as a tool for data interpretation and decision support in the context of precision fish farming.

**Methods:**

Alternative protein and oil sources, including poultry by-product meal (PBM) and natural algae oil, were explored as sustainable replacements for fishmeal (FM) and fish oil (FO). A 90-day feeding trial was conducted using rainbow trout, comparing two isoproteic, isolipidic and isoenergetic diets. The control diet contained 15% FM, 5% PBM, and 8% FO, while the test diet replaced FM with 15% PBM and 5% feather meal hydrolysate (FMH), and fully substituted FO with VeraMaris^®^ natural algae oil and rapeseed oil.

**Results:**

PBM successfully replaced FM protein without negatively affecting feed intake, growth performance or feed utilization in trout. The combination of PBM and natural algae oil was well tolerated by the trout and showed no negative effects on gut health. A detailed analysis of fatty acids in the fillet revealed that PUFAs of the n3 and n6 series were significantly higher in the PBM group than in the FM group. Values of fatty acid-related health indexes, including atherogenicity index, and thrombogenicity index, confirmed the high nutritional value of trout filet, thus representing a healthy product for human. In addition, the predictions using the FEEDNETICS™ indicated that the tested novel alternative formulations are economically viable. The validation of the model for fish growth resulted in a Mean Absolute Percentage Error (MAPE) of 8%.

**Conclusions:**

The FEEDNETICS™ application enhances our ability to optimize feeding strategies and improve production efficiency in the aquaculture industry. VeraMaris^®^ algae oil and PBM could serve as viable and sustainable raw materials for fish feed, promoting environmentally friendly aquaculture practices.

## Introduction

The aquafeed industry has grown significantly due to rising demand for fish and seafood. However, this growth has raised sustainability concern mainly due to the dependence on marine-derived ingredients such as fishmeal (FM) and fish oil (FO) whose global production is reaching its limits. As demand for aquafeed increase, the limited supply of FM and FO poses a challenge for the aquaculture industry. Therefore, finding sustainable alternatives to FM and FO is crucial for the industry’s continued growth [1, 2].

Since 1960s and 1970s, significant progress has been made in finding alternatives to FM and FO in aquafeed. These efforts have led to the partial replacement of FM and FO with terrestrial plant ingredients such as soya, rapeseed, lupins, peas, wheat, maize, and linseed which are now commonly used in aquaculture [3, 4]. While the use of herbal ingredients in aquafeeds has shown promise as a partial replacement, there are serious limitations when considered as a full replacement for FM and FO [5]. Many plant-based ingredients have relatively limited nutritional value, including unbalanced amino acid and fatty acid profiles that can lead to nutritional deficiencies in some fish species [1, 6]. In addition, certain biological components, such as antinutritional factors, may be present in plant-based ingredients that negatively impact fish performance, health and overall well-being [6, 7]. These factors highlight the need for further research and optimisation to develop sustainable and nutritionally appropriate aquafeed formulations for various fish species.

In recent aquafeed research, has shown growing interest in novel protein sources as alternatives to FM and terrestrial plant proteins. Protein sources such as processed animal proteins (PAPs) from terrestrial animals (e.g., poultry by-product meal, blood meal and feather meal), insect meal (e.g., black soldier fly and yellow mealworm), unicellular proteins (SCP) (e.g., microalgae, yeasts, bacteria and protists) and macroalgae (e.g., *Ulva*, *Gracilaria* and *Laminaria*) have emerged as potential sustainable alternatives [8, 9]. The reason for this change is their lower environmental impact compared to conventional sources. As an alternative to FM, researchers have explored various animal protein sources, including rendered animal protein products such as meat and bone meal and poultry by-product meal (PBM) [10].

Terrestrial animal proteins, including poultry by-product meal (PBM) and feather meal hydrolysate (FMH), have gained significant attention as valuable feed ingredients in aquaculture. These protein sources have been re-authorized for use in European aquafeeds and have demonstrated effectiveness as substitutes for FM in the diets of various fish species [11–13]. However, some rendered animal protein meals, such as blood meal, FMH, and meat and bone meal, may present a deficiency or excess of certain essential amino acids. This variation in amino acid profiles can lead to inconsistent performance in fish, especially when these protein sources are used alone as the primary protein in the diet [14–16]. Therefore, while PBM and FMH offer promising alternatives to FM, their amino acid composition must be carefully balanced to ensure optimal growth and health in aquaculture species.

PBM is considered one of the most promising PAPs for aquafeed formulations. It is obtained from the utilization of the non-meat parts of slaughtered poultry, such as feathers, heads, feet and internal organs [17]. PBM typically contains a protein content of 450 to 650 g/kg and is rich in most essential amino acids, with the exception of lysine and methionine [15, 18]. FMH is another alternative protein source that has gained attention in recent years. In addition, feather meal is known to be rich in certain amino acids, including cystine, threonine and arginine, which are important for fish growth and health [19]. PBM is a promising alternative and viable supplement to the diet of marine animals, which has proven its suitability for various fish such as gilthead seabream (*Sparus aurata*) [20–22], black seabass (*Centropristis striata*) [23] and red seabream (*Pagrus major*) [24]. Studies have shown that salmonids such as rainbow trout (*O. mykiss*) and Atlantic salmon (*Salmo salar*) have shown optimal growth performance when fed diets high in PBM [25–29].

When substituting FM in fish feed, it is often advisable to use a combination of different protein sources rather than relying on just one. Using terrestrial animal proteins like PBM and FMH in aquafeed not only utilizes animal by-products but also enhances the ecological efficiency of poultry production [30]. By utilizing land animal proteins in aquafeed, can improve its overall sustainability and generally have a more favorable carbon footprint and higher environmental efficiency [31].

The maximum proportion of PBM in the diet varies by fish species, PBM quality, and overall feed composition. To maintain nutritional balance, digestibility, and palatability, most studies suggest replacing only a portion of FM with PBM. However, some propose that PBM can fully replace conventional protein sources [20, 21, 32, 33]. Given these nuances, it is evident that while PBM can be a viable alternative to FM without significantly compromising growth, certain performance metrics may exhibit slight, non-significant trends that could vary depending on species, diet formulation, and other factors. We designed our experimental diets to meet the nutritional requirements of the trout, particularly focusing on a balanced amino acid profile, which likely contributed to the observed outcomes.

Ensuring the welfare of farmed fish exposed to novel feeds is currently a major concern in aquaculture and has implications for sustainability and ethical production standards [34]. Under rearing conditions, feeding poses a significant risk to fish welfare [35], as sub-optimal nutrition can disrupt physiological balance and the ability to cope with stressful situations [36]. This can have various effects, including reduced growth performance, lower feed efficiency, reduced disease resistance and potentially compromised product quality [37]. However, altered protein feeding also affects the functionality of the digestive system [38]. Fish need a healthy digestive system to realise their full potential. The intestine is the main site of nutrient digestion and absorption, while the liver is the main organ for the deposition and metabolism of nutrients.

Therefore, studying the possible effects and changes in the histomorphology of these tissues is crucial for evaluating the potential benefits of using animal proteins from land instead of FM. The histopathological effects of land-based animal proteins as a replacement for FM on liver and intestine are not well known. Studies on hybrid groupers [39, 40], *Lates calcarifer* [41] and *Lateolabrax japonicus* [42] have shown that high intakes of land-based animal proteins can induce hepatic steatosis and promote hepatic lipid vacuolization. Replacing land-based animal proteins with FM has also been associated with negative effects on gut histology [39]. Many articles have been published on the use of PBM as a sustainable protein source in FM, but most of this research has been conducted in controlled environments, such as experimental facilities, to limit variables.

In fish farming, mathematical modelling can be used to describe the complex dynamics of farming systems, taking into account factors such as fish growth rates and environmental factors. These models help fish farmers better understand their operations and predict future outcomes, such as fish growth, serving as a decision-making tool [43, 44]. Significant progress has been made in developing mathematical models to support fish farming practices [45, 46]. Recently, FEEDNETICS™ (Software; FEEDNETICS, 2022, Olhão, SPAROS), a mechanistic, nutrient-based tool designed to assist with data interpretation and decision-making in precision fish farming, was developed. This model provides a range of applications for fish farming, with the objective of improving efficiency and productivity in various aspects of the industry [47]. FEEDNETICS serves as a computational tool specifically developed for the analysis and optimization of feed formulations and feeding strategies in aquaculture research.

Unlike previous studies that focused solely on PBM as a replacement for FM, our study investigates the combined use of PBM and FMH along with VeraMaris^®^ algae oil. The inclusion of FMH is intended to complement the amino acid profile provided by PBM, addressing the deficiency in lysine and methionine. VeraMaris^®^ algae oil serves as an alternative source of omega-3 fatty acids, which are crucial for fish growth and health. In the present study, we therefore investigated the effects of completely replacing FM with PBM and FMH, alongside the full substitution of FO with VeraMaris^®^ natural algae oil and rapeseed oil in the diet of rainbow trout (*O. mykiss*). The impact on fish growth performance, gut and liver morphology, production of volatile fatty acids in the gut, lipid metabolism indices, and fatty acid profile in fillets was thoroughly examined. We also validated the data from the in vivo experiments using a nutrient-based FEEDNETICS™ model, which can be used as a tool for data interpretation and decision support in the context of efficient fish farming.

## Materials and methods

### Experimental diets

The selection of alternatives to FM and FO ingredients was made within the framework of the circular economy, which aims to reduce waste throughout the agri-food value chain and promote sustainability both within the current constraints and the expected regulatory framework. The feeds were developed in accordance with the nutritional requirements of rainbow trout (*O. mykiss*) [48] and produced by extrusion at the SPAROS facilities in Olhão, Portugal.

Two experimental feeds were produced, designated as the control feed (FM) and test feed (PBM). The feeds were carefully formulated to ensure that they contained the same levels of nitrogen, lipids and energy to ensure a fair comparison of their effects on the growth performance of rainbow trout. The FM diet contained a higher proportion of FM, specifically 15%, along with 5% poultry meal (PM) and other protein sources. In contrast, the PBM test diet was formulated to replace FM with 15% PM and included an additional 5% FMH to balance the amino acid profile. Notably, the PBM diet, did not include any FO. Instead, FO was replaced by 5.35% algae oil (VeraMaris^®^ algae oil) and an increased proportion of rapeseed oil. The algae oil VeraMaris^®^ was supplied by VeraMaris^®^ V.O.F., a joint venture between DSM and Evonik, specializing in producing omega-3 fatty acids EPA and DHA from natural marine algae for animal nutrition. This innovative inclusion aimed to provide a sustainable source of omega-3 fatty acids without relying on traditional FO sources.

Detailed information on the formulation and approximate composition of the two diets can be found in Table 1. The feed preparations were carried out using an extrusion process at the SPAROS LDA trial facility. In this process, all ground ingredients were carefully mixed with oil, and water was added to achieve the required moisture content for extrusion. The mixture was then passed through an extruder at high temperature and pressure, which resulted in the expansion of the feed material and the formation of pellets. The extruded pellets were dried at 50 °C for 48 h to remove excess moisture.

**Table 1:** Ingredients and proximate composition of the experimental feeds

	Experimental diets	
Ingredients (% as feed):	FM	PBM
Fishmeal Super Prime	15.0	0
Poultry meal	5.0	15.0
Feather meal hydrolysate	0	5.0
Fish protein hydrolysate	3.0	3.0
Fish oil	8.0	0
Rapeseed oil	13.1	16.75
Algae oil VeraMaris^®^	0	5.35
Porcine blood meal	2.0	2.0
Insect meal (*Hermetia illucens* larvae)	2.0	2.0
Soy protein concentrate	12.6	12.0
Wheat gluten	9.1	7.1
Corn gluten meal	5.0	5.0
Soybean meal	6.5	6.5
Wheat meal	12.15	12.73
Whole peas	3.0	3.0
Vitamin and mineral premix	1.0	1.0
Choline chloride 50%	0.2	0.2
Antioxidant	0.2	0.2
Sodium propionate	0.1	0.1
MAP (Monoammonium phosphate)	1.2	1.65
L-Lysine HCl 99%	0.4	0.8
DL-Methionine	0.1	0.27
L-Taurine	0.2	0.2
Soy lecithin	0.15	0.15
**Proximate composition (% as feed)**:		
Crude protein	43.0	43.0
Crude fat	23.0	23.0
Fiber	1.7	1.7
Starch	11.6	11.8
Ash	5.8	5.3
Gross energy (MJ/kg as feed)	22.6	22.6

### Chemical analysis of feeds

The chemical analysis of the feeds was carefully performed, with measurements taken in triplicate to ensure accuracy and reliability. Various nutritional components were evaluated, including dry matter (DM, AOAC #930.15), crude protein (CP, AOAC #2001.11), crude fiber (AOAC #978.10), ash (AOAC #942.05), and ether extract (EE, AOAC #920.39) content. These determinations were performed in accordance with the established standard procedures of the Association of Official Analytical Chemists [49], with emphasis on the rigor and precision of the analytical methods.

Gross energy assessment was performed using an adiabatic calorimetric bomb (C7000; IKA, Staufen, Germany), which provides information on the total energy content of the feeds. The comprehensive composition of the feeds, which includes these important nutritional components, is shown in Table 1.

In addition to the basic nutritional components, the detailed composition of amino acids, fatty acids and minerals in the two meals was carefully analyzed (Tables 2, 3 and 4). The protocols used for those specific analysis have been described in detail in [50].

**Table 2:** Amino acid composition (g/100 g dry matter) of the experimental feeds

	FM	PBM
Essential amino acids (EAA)		
Arginine	2.3	2.4
Histidine	1.0	0.9
Isoleucine	1.6	1.6
Leucine	3.4	3.3
Lysine	2.6	2.6
Threonine	1.5	1.6
Tryptophan	0.5	0.4
Valine	1.9	1.9
Methionine	0.9	0.9
**Non-essential amino acids (NEAA)**		
Cysteine	0.6	0.6
Methionine + Cysteine	1.4	1.5
Phenylalanine	2.0	1.9
Tyrosine	1.4	1.3
Phenylalanine + Tyrosine	3.4	3.3
Asparagine	3.4	3.3
Glutamine	8.2	7.6
Alanine	2.2	2.1
Glycine	2.1	2.5
Proline	2.7	3.0
Serine	1.9	2.2
Tubulin associated unit	0.3	0.3

**Table 3:** Fatty acid composition (g/100 g dry matter) of the test feeds

	FM	PBM
Myristic acid (C14)	0.7	0.2
Palmitic acid (C16)	2.5	2.5
Octadecanoic acid (C18)	0.5	0.4
Oleic acid (C18:1n9)	8.4	9.3
Linoleic acid (C18:2n6)	2.9	3.6
Alpha-Linolenic acid (C18:3n3)	1.2	1.4
Arachidonic acid (C20:4n6)	0.1	0.1
Eicosapentaenoic acid (C20:5n3)	1.5	0.8
Docosahexaenoic acid (C22:6n-3)	1.3	2.0
Eicosapentaenoic acid + Docosahexaenoic acid	2.8	2.8

**Table 4:** Mineral profile (mg/kg dry matter) of the experimental feeds

	FM	PBM
Ca	0.6	0.5
Na	0.3	0.2
Mg	0.1	0.1
K	0.7	0.6
Cu	10.7	10.9
Fe	250.6	153.7
I	3.6	3.4
Mn	26.7	26.5
Se	0.6	0.4
Zn	106.8	102.2

### Fish rearing conditions

The feeding trial with rainbow trout (*O. mykiss*) was carried out at the agricultural company “Fattoria del Pesce” in Cerano, Novara, Italy (authorization of the Italian Ministry of Health no. 143810 of 19/03/2019). At the beginning of the experiment, about 2,908 juvenile rainbow trout (initial body weight, IBW = 27.5 ± 0.3 g; mean ± standard deviation, SD) were randomly distributed in 4 rectangular outdoor tanks of 10 m^3^ each with 727 fish/tank (initial density = 20 kg/m^3^) and acclimatized for one week. During the acclimatization week, the fish were kept under a natural photoperiod and fed a commercial feed containing approximately 45% crude protein and 20% crude lipid. The primary protein source in the commercial feed was fishmeal, while fish oil was the primary lipid source.

After the acclimatization period, each feed was distributed for 90 days to two tanks with an initial biomass of approximately 20 kg of rainbow trout. Trout were hand-fed either FM or PBM feed once a day, 7 days a week with a daily meal (between 08:00 and 10:00). The feeding rate was set at a ratio of 1.5-2.0% of the total biomass in each tank and was adjusted throughout the trial based on water temperature, fish growth and feed intake to ensure that all the feed was consumed. The feeding trial began on 1 December 2022 and was conducted over 90 days. Throughout the experiment, parameters such as temperature (between 9.4 and 12.4 °C), pH, dissolved oxygen concentration, total ammonia-nitrogen, and salinity were continuously monitored to maintain optimal environmental conditions for the trout. The biomass in each tank was determined by weighing all fish individually on a monthly basis. Fish mortality in the tanks was monitored daily.

Due to logistical constraints and the commercial nature of the farm setup, it was feasible to allocate only two tanks for each dietary treatment. While the number of tanks was limited, the large number of fish per tank provided a substantial sample size at the individual fish level, allowing for a detailed analysis of the dietary effects.

### Samples collection

At the end of the 90-day feeding trial, 32 fish per feeding group (16 fish/tank) were sacrificed by an overdose of anesthetic (100 mg/L tricaine methanesulfonate, MS222) (Sigma Aldrich, Saint-Louis, MO, USA). After measuring the final body weight BW (g) and final standard-length SL (cm) for the calculation of key performance indicators, six fish per tank (12 per diet) were used for sampling of liver and entire intestine. A small piece of the liver, anterior intestine (AI) and posterior intestine (PI) (0.5 cm) was fixed in a solution of neutral buffered formalin (NBF, 10%) to perform histological analysis, while the fecal samples were collected and stored at -80 °C for short‑chain fatty acid (SCFA) analysis. Fillets were also collected and pooled (6 fish/tank, 12 /diet) to analyze proximate composition and fatty acid profile and stored at -80 °C.

### Growth performance

To calculate the growth indices, all fish were weighed individually each month. The following indices were calculated for each feed group:

Survival rate (SR, %) **=**$\:\:100\:\times\:\left[\frac{\text{F}\text{i}\text{n}\text{a}\text{l}\:\text{f}\text{i}\text{s}\text{h}\:\text{n}\text{u}\text{m}\text{b}\text{e}\text{r}}{\text{I}\text{n}\text{i}\text{t}\text{i}\text{a}\text{l}\:\text{f}\text{i}\text{s}\text{h}\:\text{n}\text{u}\text{m}\text{b}\text{e}\text{r}}\right]$

Weight gain (WG, g) = Average Final body weight (FBW, g)$\:\:-\:\text{A}\text{v}\text{e}\text{r}\text{a}\text{g}\text{e}\:\text{I}$nitial body weight (IBW, g)

Specific growth rate (SGR, % day − 1) = $\:100\:\times\:\:\frac{\text{l}\text{n}\:\text{W}\text{t}-\text{l}\text{n}\:\text{W}\text{i}}{\text{t}}$

Condition factor (CF, %) = $\:100\:\times\:\left[\frac{\text{b}\text{o}\text{d}\text{y}\:\text{w}\text{e}\text{i}\text{g}\text{h}\text{t},\:\:\:\left(\text{g}\right)}{\text{b}\text{o}\text{d}\text{y}\:\text{l}\text{e}\text{n}\text{g}\text{t}\text{h}\:\left(\text{c}\text{m}\right)3}\right]$

Feed Conversion Ratio (FCR) = cumulative feed per fish / Av. Weight gain.

Where, Cumulative feed per fish = sum of the feed per fish during the trial.

Feed per fish = feed given / nº of fish (per day).

And Av. Weight gain = Av. Final weight – Av. Initial weight.

Where, Wt is final body weight (g), Wi is initial body weight (g), ln is natural log, and t is experimental duration in days.

### Histology of the intestine and liver

For the histological evaluation, the anterior and posterior intestinal segments as well as the liver of 12 fish per diet were removed, dehydrated and embedded in paraffin using standard techniques. Subsequently, the intestinal samples were cut into 5 μm cross-sections using a microtome (Leica RM2245, Leica Biosystems, Milan, Italy) and stained with hematoxylin-eosin (H&E) for examination under a light microscope (Zeiss Axiophot microscope, Milan, Italy). Tissue morphology was analyzed using a CMOS Discovery C30 digital camera mounted on the microscope. Images were captured using an Olympus IX51 light microscope and processed using Fiji software, an open-source Java-based imaging program (https://fiji.sc/, accessed 15 January 2024). A semi-quantitative assessment of inflammation and accumulation of fatty deposits in the liver was performed to verify liver health, using a scale from 1 to 5, which corresponds to the categorization for histological features proposed by [51–53]. Semi-quantitative analysis was performed on a randomized sequence of samples. For each dietary group (12 samples), these features were assessed in 9 randomly selected areas and in 3 sections. The histological assessment of the intestine included the measurement of 4 main morphological criteria as described by [54]. These criteria were villus height (ViH), villus width (ViH), lamina propria width (LPW) and submucosa width (SW).

### Quantitative and qualitative analysis of short-chain fatty acids (SCFAs) in feces

The analysis of fecal short-chain fatty acids (acetate, propionate, iso-butyrate and butyrate) included both qualitative and quantitative assessments using a modified method of [55]. First, 0.5 g of fecal material was mixed with 5 mL of HPLC PLUS water in a falcon tube and vortexed for three minutes. This mixture was then centrifuged at 4,000 rpm for 30 min. The clear liquid above the sediment (the supernatant), was passed through a 0.22 µ PTFE syringe filter for further purification. A high-performance liquid chromatography (HPLC) system with UV-VIS detection, a Shimadzu model from Milan, Italy, was used for the analysis.

The HPLC system comprised two LC-20AD pumps, a CBM-Alite controller, a DGU-20A5 degasser and an automatic sample injector. For the chromatographic process, an Ascentis Express 90 A C18 column with an inner diameter of 4.6 mm, a length of 150 mm and a particle size of 2.7 μm was used, which was manufactured by Merck KGaA in Darmstadt, Germany. The separation was performed in isocratic mode with a mobile phase of 0.005 M sulfuric acid (H_2_SO_4_) flowing at 0.6 mL/min and a column temperature of 60 °C. Each sample injection was 10 µl and UV-VIS detection was performed at a wavelength of 210 nm using LC Solution Software (S/N L52405100502LG) from Shimadzu, Milan, Italy. To identify the SCFAs, their retention times were compared with those of known standards. For quantification, a calibration curve method was used for different concentrations and the results were expressed in millimoles/L.

### Analyses of the fatty acid composition of fish fillet

To assess the fatty acid profile, total lipids were first extracted from 2 g of minced, moist muscle fillet (24 samples in total, 12 fish per diet and 6 fish per replicate). The total lipid content was extracted with a chloroform/methanol solution (2:1, v/v) according to the Folch method [56]. Subsequently, the total lipids obtained were used to produce fatty acid methyl esters (FAMEs) for the evaluation of the FA profile according to the method described in [57]. Specifically, 2 ml of methanol-sulfuric acid solution (9:1, v/v) was added to each oil sample and heated at 100 °C for 1 h.

The FAMEs were then analyzed using a Trace 1310 gas chromatograph (GC) (Thermo Fisher Scientific, Milan, Italy) equipped with a flame ionization detector (FID). FAMEs were separated using a fused silica capillary column (Omegawax 250; Supelco, Bellefonte, PA, USA) measuring 30 m × 0.25 mm (length × inner diameter) coated with a 0.25 μm thick layer. The temperature of the column was initially set to 100 °C for 5 min, followed by a ramp from 100 to 240 °C at a rate of 4 °C/min and a final isothermal hold at 240 °C for 20 min. The temperatures of the injector and detector were maintained at 250 °C. The injection volume and split ratio were 0.5 µl and 1:50, respectively, and helium (He) was used as the carrier gas at a flow rate of 1 ml/min. Data acquisition and processing were performed using Chromeleon™ software (Thermo Fisher Scientific, Milan, Italy).

The FAs in the fish samples were identified by comparing the relative retention times of the FAMEs with those of a mixed standard solution (37 mixed FAMEs; Supelco, Inc., Bellefonte, PA, United States) analyzed under the same analytical conditions. The quantification was performed by integrating the individual peaks, with the percentage area fractions calculated by considering the sum of all identified FAMEs as 100 g. FA concentrations were then expressed in g/100 g of the sample.

### Indices for the nutritional quality of fillets and for the estimation of elongase and desaturase activity

Nutritional indices considering the different fatty acids in terms of their different contribution to the promotion or prevention of cardiovascular disease were assessed. The dietary indices for atherogenic (AtheroI) and thrombogenic (TI) fatty acids were calculated using the equations of Ulbricht and Southgate [58], while the ratio between hypocholesterolemic and hypercholesterolemic fatty acids (h/H) was calculated using the equation of [59] as follows:

1. Atherogenicity index [AtheroI = (C 12:0 + (4 x C 14:0) + C 16:0)/ (Ʃ Monounsaturated Fatty Acids (MUFAs) + n-6 PUFAs + n-3 PUFAs)];
2. Thrombogenicity index [TI = (C 14:0 + C 16:0 + C 18:0)/ (0,5 x Ʃ MUFAs + 0,5 x n-6 PUFAs + 3 x n-3 PUFAs) + (n-3 PUFAs/ n-6 PUFAs)];
3. Hypocholesterolemic/hypercholesterolemic ratio (h/H = (C18:1n9 + C18:2n6 + C20:4n6 + C18:3n3 + C20:5n3 + C22:5n3 + C22:6n3) / (C14:0 + C16:0)].

In addition, the peroxidation index (PI), which expresses the peroxidation susceptibility and peroxidative lipid damage for a given phospholipid membrane, was calculated using the equation of [60] as follows:

PI = (% dienoic × 1) + (% trienoic × 2) + (% tetraenoic × 3) + (% pentaenoic × 4) + (% hexaenoic × 5).

To estimate the activities of the enzymes involved in the elongation and desaturation of fatty acids, the ratio between product and precursor was calculated using the following equations from [61]:

4. Thioesterase = C16:0/ C14:0;
5. Elongase = C18:0/ C16:0;
6. ∆9 desaturase (C16) = [(C16:1)/ (C16:1 + C16:0)]x100;
7. ∆9 desaturase (C18) = [(C18:1)/ (C18:1 + C18:0)]x100;
8. ∆9 desaturase (C16 + 18) = [(C16:1 + C18:1)/ (C16:1 + C16:0 + C18:1 + C18:0)]x100;
9. ∆5 + ∆6 desaturase (n6) = [(C20:2n6 + C20:4n6)/ (C18:2n6 + C20:2n6 + C20:4n6)]x100;
10. ∆5 + ∆6 desaturase (n3) = [(C20:5n3 + C22:5n3 + C22:6n3)/ (C18:3n3 + C20:5n3 + C22:5n3 + C22:6n3)]x100.

### Validation of the test results with FEEDNETICS™

The results from the in vivo experiment were validated using the nutrient-based model FEEDNETICS™ (FEEDNETICS Software, SPAROS, Olhão, 2022), as described in [47]. In the present study, the most effective feed for rainbow trout was determined. Data collected from the feeding trial, including fish growth performance, feed composition and feeding regimes, were organized and prepared for analysis within the FEEDNETICS™ application.

### Validation of the FEEDNETICS™ model under farm conditions

To confirm the accuracy of the predicted fish growth performance, the FEEDNETICS™ model was validated using data from our experiment. This validation approach was developed to confirm that the predictions of the model closely matched the observed data. In this way, confidence in the model’s ability to accurately estimate the impact of nutrition is strengthened and any unaccounted-for elements affecting fish performance can be uncovered.

The validation was conducted at the tank level, with input data coming from experimental measurements. The measured values included the daily number of fish, initial weight, water temperature, feed quantity and proximate composition. The qualitative assessment compared the model’s predictions, particularly for fish growth and feed intake, with the observed experimental data. For the quantitative analysis, the mean absolute percentage error (MAPE) was calculated to evaluate the accuracy of the model’s predictions against the observed data on specific sample days. This comprehensive validation process confirms the reliability of the model in predicting fish performance under a wide range of husbandry conditions through the following:

$$\mathrm{MAPE}\,(\%)=\frac{100}n\sum\nolimits_{i=1}^n(\frac{Pi-Oi}{Oi})$$

Where, n is the number of predicted-observed value pairs; Pi is the predicted value; Oi is the observed value.

### Data input into FEEDNETICS™

The data recorded during the trout experiment was uploaded to the FEEDNETICS™ application model. An overview of the input data used to run this model can be found in Table 5. We used a baseline scenario with an average fish body weight of 28 g, 2,908 fish and a monthly mortality rate recorded throughout the experiment. The validation was conducted beyond the 90-day trial period. Specifically, predictions were run to compare the model’s predicted outcomes with the observed data from the experiment. This validation process was designed to access how accurately the FEEDNETICS™ model could predict the growth and development of rainbow trout based on different feed formulations over the trial period. The observed monthly average water temperature data was used as an input for the predictions.

**Table 5:** Feeding trial data for rainbow trout (*O*. *mykiss*) used in FEEDNETICS^™^ validation scenarios

Category	Inputs	
Stock (farming conditions)	Species: Rainbow trout (*Oncorynchus mykiss*) Initial weight (g): 28 g Initial number of fish: 2,908 Tank/cage volume (m^3^): 2.5 Start date: 01/12/2022 Production period (months): 3 Number of dead fish: Recorded from the experiment	
Feed proximate composition	FM	Proximate composition: analyzed values Amino acid profile: estimated values Fatty acid profile: default values Digestibility: default values
	PBM	
Feeding records	Daily ration (kg) Number of meals: 1 meal/day Time of the 1st meal: 9 AM Feed waste: 0%	
Temperature	Monthly water temperature: 10–12 ^o^C	
Cost & revenues	Price: default price 1.2 €/kg of feed	

The proximate composition of the feed, including crude protein, crude fat, ash, fiber, phosphorus and gross energy, was uploaded to FEEDNETICS™ application model. The proximate composition of the feed was defined based on the analyzed values, while the amino acid profile of the experimental feed was defined based on the values from the feed ingredient composition. In addition, the fatty acid profiles, digestibility data and pricing were included in the model as standard variables for rainbow trout.

In addition to the proximal composition of the feed, it was also necessary to enter information on the daily feeding protocol, such as the daily ration (in kg), the number of meals into which the daily ration was divided, the time of the first meal (in hours), the intervals between meals (in hours) and the uneaten feed (in %).

Once all the required data had been entered into the FEEDNETICS™ application model, predictions were generated and compared with those of our experimental study.

### Sstatistical analysis

All statistical analyses were performed using PAST v3 software [62], with significance set at *p* < 0.05. The normality of the data set was checked using the Shapiro-Wilk test. Inequalities between the mean values were analyzed using the Student’s t-test or the Mann-Whitney test if the data did not correspond to the assumption of a normal distribution. Given the limitation of using only two tanks per dietary treatment due to logistical constraints, statistical analyses were conducted at the individual fish level to maximize the robustness of the results. By analysing a large number of fish, we aimed to ensure the reliability of the statistical evaluation despite the limited tank replicates, focusing on detecting differences at the individual fish level to draw meaningful conclusions.

## Results

### Growth performance and feed utilization

The fish quickly acclimatized to the experimental feeds from the start of the trial. The detailed results of growth performance and feed utilization during the 90-day feeding trial are shown in Table 6. The mortality rate remained consistently low throughout the trial and was around 5% in both groups of fish. No significant differences in mortality rates were observed between the trout fed the different diets. High survival rates (> 97%) were observed for both feed treatments, with no significant differences between fish groups (*p* > 0.05). Final body weight (FBW) was 170.80 ± 7.61 g and 166.91 ± 6.62 g for fish fed FM and PBM diets, respectively, reflecting an individual weight gain of more than 140 g compared to initial body weight (IBW), with no significant differences.

**Table 6:** Indicators of growth performance and feed utilization of rainbow trout fed the experimental diets for 90 days. (*n* = 2 tanks and 32 fish/tank)

Growth Parameters	FM	PBM	*p*-Value
SR (%)	96.88 ± 1.28	97.68 ± 1.30	0.70
IBW (g)	27.5 ± 7.61	27.5 ± 6.24	0.61
FBW (g)	170.80 ± 7.61	166.91 ± 6.62	0.70
WG (g)	143.30 ± 7.61	139.41 ± 6.62	0.70
SGR (% day^−1^)	2.03 ± 0.04	2.00 ± 0.08	0.77
FCR	1.33 ± 0.05	1.37 ± 0.11	0.74
TL (cm)	22.91 ± 0.34	22.70 ± 0.29	0.65
SL (cm)	20.89 ± 0.33	20.66 ± 0.28	0.58
CF	1.40 ± 0.02	1.41 ± 0.02	0.78

Values are presented as mean ± SEM of two replicate groups

*Abbreviations*: *IBW* Initial body weight, *FBW* Final body weight, *WG* Weight gain, *SGR* Specific growth rate, *FCR* Feed conversion ratio, *TL* Final total length, *SL* Final standard length, *CF* Condition factor

SGR was numerically slightly higher in the FM group (2.03 ± 0.04% day^−1^) than in the PBM group (2.00 ± 0.08% day^−1^), but again no significant differences in WG and SGR were observed between the fish fed different diets. FCR was similar between the groups, with the FM-fed fish showing a slightly lower value (1.33 ± 0.05). There were no significant differences in the total length of the fish (22.91 ± 0.34 cm and 22.70 ± 0.29 cm respectively) and the standard length between the two experimental groups. The fish fed with the different diets were healthy and showed no visible lesions on their external and internal organs. Analysis of the Fulton’s condition factor (CF) revealed no significant differences (1.40 ± 0.02 vs. 1.41 ± 0.02) between the FM and PBM fish groups.

### Histomorphology of the anterior and posterior intestine

**Fig. 1:**
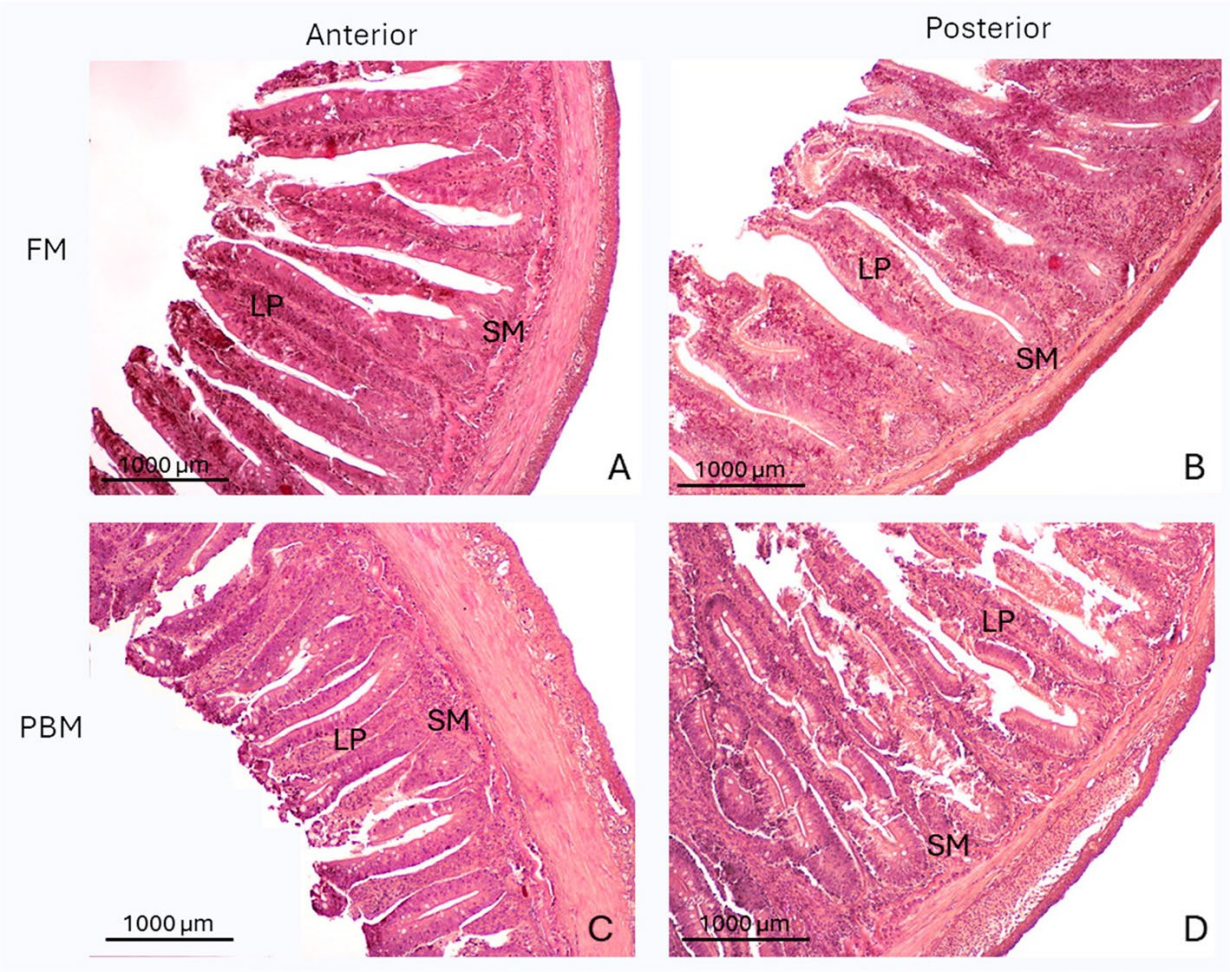
Standard hematoxylin and eosin (H&E) staining of anterior and posterior intestinal sections of FM (panels A, B) and PBM (panels C, D). SM, submucosa; LP, lamina propria; FM, fishmeal; PBM, Poultry by-product meal. Scale bar: 1000 μm

Gross visual examination of rainbow trout revealed no significant differences in the anterior intestine (AI) and posterior intestine (PI) between fish fed FM and PBM. However, histological analysis revealed clear differences in the structure of the intestinal tissue. Samples from PBM-fed fish showed well-organized and preserved tissue with intact mucosal folds, indicating no signs of damage or inflammation compared to FM-fed fish. The mucosal folds in the AI were more complex and numerous than in the PI, with simple folds interspersed with complex folds in the distal part. The intestinal tissue showed a typical histological organization, with a columnar epithelial layer supported by connective tissue and surrounded by the muscle layer. The epithelial cells were mainly enterocytes with acidic microvilli, basal basophilic nuclei, eosinophilic cytoplasm and - depending on the diet - varying amounts and sizes of clear supranuclear vacuoles. The morphometric measurements of the fish intestines are shown in Table 7. The height of the intestinal villi in the anterior intestine (AI) was significantly greater (*p* < 0.05) in fish fed the PBM diet compared to those fed the FM diet, as shown in Table 7; Fig. 1. There was no significant difference (*p* > 0.05), the villi width, the lamina propria width (LPW) and the submucosa width (SW) in the AI between the two dietary groups. In the posterior part of the intestine, villus length, width and LPW did not differ significantly (*p* > 0.05) between the two diet groups. However, the thickness of the submucosa was significantly increased in the PBM diet group compared to the FM diet group (*p* < 0.05), indicating a possible inflammatory response (Fig. 1).

**Table 7:** Intestinal morphological parameters of the anterior and posterior intestine of rainbow trout fed two experimental diets (*n* = 12)

	Diet		
	FM	PBM	*p*-value
Anterior Intestine			
Villus height (µm)	285.7 ± 5.56	320.6 ± 9.77	*<*0.003
Villus width (µm)	84.45 ± 4.26	79.34 ± 4.03	0.39
Lamina propria width (µm)	46.12 ± 2.79	50.42 ± 2.91	0.29
Submucosa width (µm)	81.65 ± 2.43	84.98 ± 4.49	0.51
**Posterior Intestine**			
Villus height (µm)	655.8 ± 30.99	605.7 ± 33.15	0.27
Villus width (µm)	120.8 ± 5.69	135.3 ± 6.66	0.10
Lamina propria width (µm)	49.52 ± 3.2	53.54 ± 3.72	0.42
Submucosa width (µm)	67.17 ± 3.56	88.16 ± 3.09	*<*0.0001

Values are presented as mean ± standard error

### Histomorphology of the liver

**Fig. 2:**
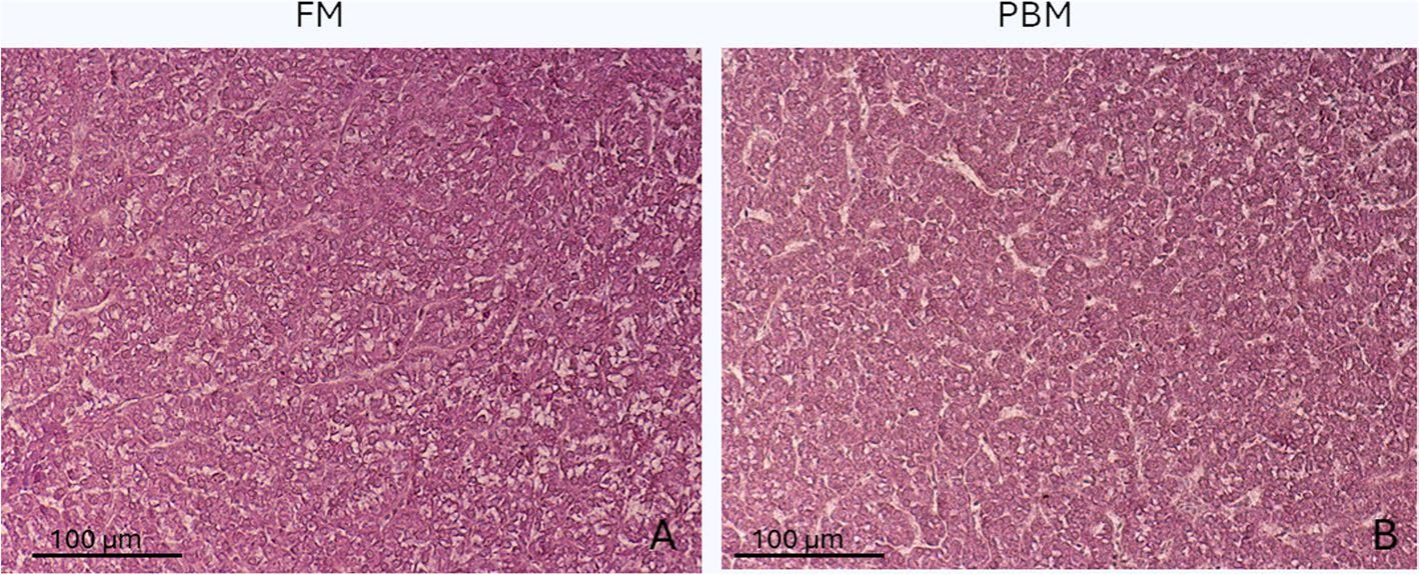
Standard hematoxylin and eosin (H&E) staining of liver from FM (**A**) and PBM fish (**B**). Scale bar, 100 μm

In both dietary groups, the livers showed normal parenchymal architecture. The structure of the hepatic cord remained regular, with a clear demarcation by sinusoids and continuous bile ducts (Fig. 2). The hepatocytes had a polyhedral shape and showed varying degrees of vacuolization in their cytoplasm. They were organized in anastomosed plates subdivided by sinusoidal capillaries leading to central veins. There were no signs of inflammation or lymphocyte infiltration in any of the diets tested. Interestingly, the diets had different effects on the hepatocytes.

**Fig. 3:**
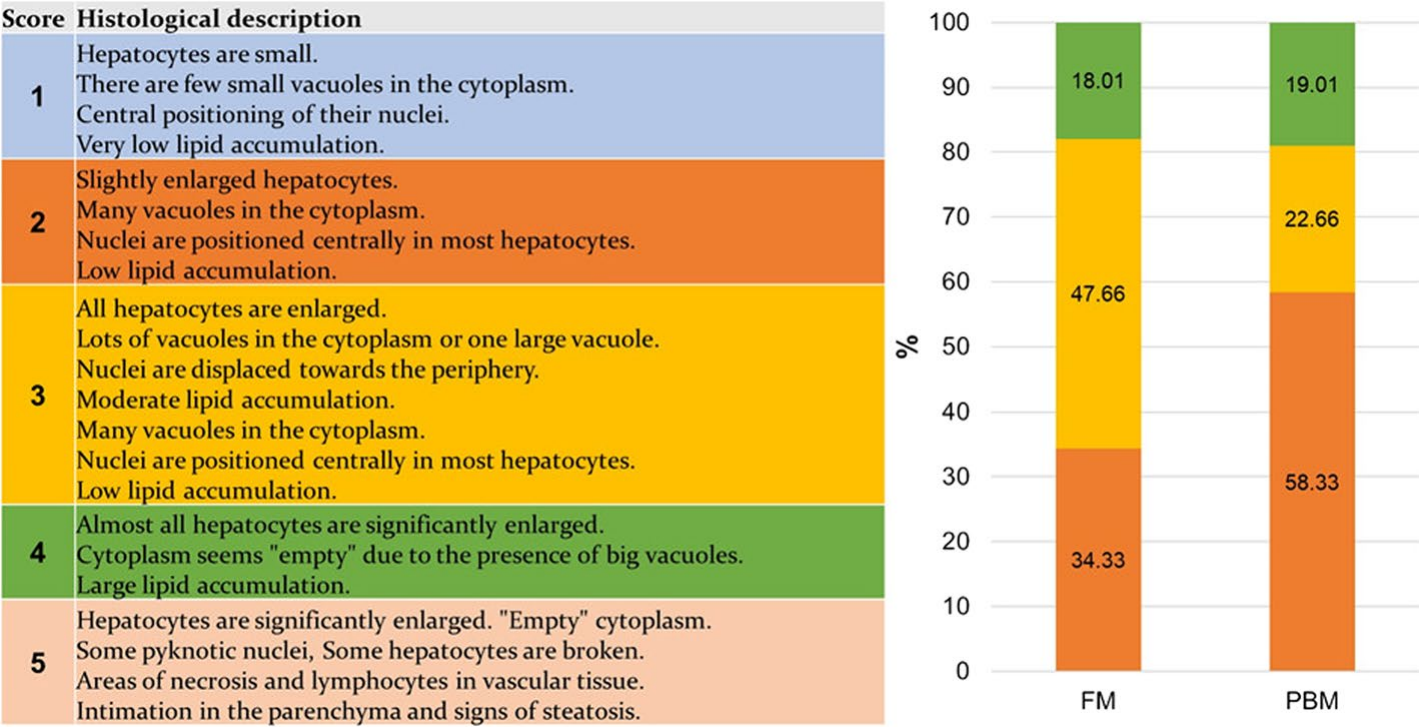
Semi-quantitative evaluation of liver fat accumulation in rainbow trout fed the experimental diets FM and PBM. The liver values are given in percentage and were measured in 6 fish/diet group

Fish fed the FM diet showed moderate lipid accumulation, which led to a displacement of the nuclei at the periphery of the hepatocytes (scoring class 2 and 3; Fig. 3). In contrast, the inclusion of 20% PM in the PBM diet had a marked effect on the accumulation of lipid deposits within hepatocytes and resulted in less lipid accumulation in the liver (classifications 2; Fig. 3). This suggests that the PBM group had less lipid deposition and accumulation in their hepatocytes. Interestingly, fish fed the PBM diet showed slight vacuolization of cells in certain regions (Fig. 2). However, this minimal vacuolization had no significant effect on the overall cell structure or physiological morphology of the organs.

### Volatile SCFAs in fecal samples

The content of SCFAs, including acetate, propionate, butyrate and isobutyrate, was measured by HPLC in the fecal samples of fish fed two different experimental diets. Table 8 shows the concentrations of these SCFAs in the samples. Interestingly, the group of fish fed the 20% PBM diet showed a remarkable increase in acetate (18.73 mmol/L) and butyrate (0.91 mmol/L) compared to the fish fed the FM diet. In contrast, the levels of propionate and isobutyrate in the fecal samples showed no significant differences between the two experimental groups, as shown in Table 8.

**Table 8:** Volatile SCFA content in fecal samples from two experimental groups

	FM	PBM	*p*-value
**Acetate (C2:0)**	17.56 ± 0.30	18.73 ± 0.20	*<0.01*
**Propionate (C3:0)**	3.62 ± 0.22	3.67 ± 0.06	ns
**Butyrate (C4:0)**	0.52 ± 0.01	0.91 ± 0.04	*<0.0001*
**Isobutyrate (C4:0)**	0.36 ± 0.01	0.41 ± 0.01	*<0.01*

Values are expressed in millimoles per litre (mmol/L), mean ± SEM (*n *= 12)

### Fatty acid composition of fish fillets

The composition of the fatty acids in the muscle of the rainbow trout is shown in Table 9. The composition was significantly influenced by diet, with significant variations observed for individual FA groups, with the exception of the proportion of heptadecenoic acid (C17:1) and dihomo-γ-linolenic acid (C20:3n6).

**Table 9:** Fatty acid composition (g/100 g FAMEs) of the fillets of rainbow trout fed with the two experimental diets

	FM	PBM	*p*-value
**C12:0**	0.23 ± 0.01	0.20 ± 0.00	*<0.01*
**C14:0**	2.36 ± 0.05	0.78 ± 0.01	*<0.0001*
**C14:1**	0.02 ± 0.00	0.01 ± 0.00	*<0.001*
**C15:0**	0.17 ± 0.00	0.13 ± 0.00	*<0.0001*
**C16:0**	15.00 ± 0.12	13.25 ± 0.12	*<0.0001*
**C16:1**	3.02 ± 0.09	0.83 ± 0.02	*<0.0001*
**C17:0**	0.16 ± 0.00	0.11 ± 0.00	*<0.0001*
**C17:1**	0.07 ± 0.00	0.07 ± 0.00	ns
**C18:0**	3.59 ± 0.07	3.01 ± 0.05	*<0.0001*
**C18:1n9**	34.00 ± 0.16	37.09 ± 0.17	*<0.0001*
**C18:1n7**	3.00 ± 0.03	2.28 ± 0.02	*<0.0001*
**C18:2n6**	12.70 ± 0.12	14.51 ± 0.14	*<0.0001*
**C18:3n6**	0.15 ± 0.01	0.18 ± 0.01	*<0.05*
**C18:3n3**	2.97 ± 0.06	3.30 ± 0.05	*<0.05*
**C20:0**	0.20 ± 0.01	0.26 ± 0.01	*<0.0001*
**C20:1n9**	1.33 ± 0.03	1.24 ± 0.03	*<0.05*
**C20:2n6**	0.57 ± 0.02	0.65 ± 0.02	*<0.01*
**C20:3n6**	0.37 ± 0.0	0.33 ± 0.02	ns
**C20:4n3**	0.85 ± 0.04	1.05 ± 0.04	*<0.001*
**C20:4n6**	0.13 ± 0.01	0.16 ± 0.01	*<0.01*
**C20:5n3**	4.81 ± 0.11	2.88 ± 0.06	*<0.0001*
**C22:0**	0.11 ± 0.01	0.13 ± 0.01	*<0.05*
**C22:1n9**	0.28 ± 0.01	0.32 ± 0.01	*<0.01*
**C22:5n3**	1.29 ± 0.03	0.75 ± 0.02	*<0.0001*
**C22:6n3**	11.91 ± 0.14	15.34 ± 0.11	*<0.0001*
**C24:1n9**	0.70 ± 0.03	1.15 ± 0.04	*<0.0001*

Values are presented as mean ± standard error (*n *= 12). The concentration of fatty acids was expressed in g/100g, where 100g is the sum of the areas of all identified FAME

The distribution of the fatty acid classes in the fillets is summarized in Table 10. The sum of saturated fatty acids (SFAs) was significantly lower in fish fed PBM than in fish fed FM, which had the highest content (21.82%). There was no significant difference in the sum of monounsaturated fatty acids (MUFAs) between the PBM and FM groups. However, the sum of polyunsaturated fatty acids (PUFAs), EPA + DHA and PUFAs of the n3 and n6 series were significantly higher in the PBM group than in the FM group. The n3/n6 PUFA ratio did not differ significantly between the two groups.

**Table 10:** Fatty acid classes and nutritional indices of rainbow trout fillets fed with the two experimental diets

	FM	PBM	*p*-value
**∑SFA**	21.82 ± 0.12	17.86 ± 0.16	*<0.0001*
**∑MUFA**	42.43 ± 0.26	42.99 ± 0.19	ns
**∑PUFA**	35.75 ± 0.21	39.15 ± 0.13	*<0.0001*
**∑n3-PUFA**	21.83 ± 0.25	23.32 ± 0.15	*<0.0001*
**∑n6-PUFA**	15.22 ± 0.12	16.58 ± 0.12	*<0.0001*
**∑n3/n6**	1.44 ± 0.02	1.41 ± 0.02	ns
**∑EPA + DHA**	16.72 ± 0.22	18.21 ± 0.14	*<0.0001*
**AtheroI**	0.31 ± 0.00	0.20 ± 0.00	*<0.0001*
**TI**	0.22 ± 0.00	0.17 ± 0.00	*<0.0001*
**h/H ratio**	3.91 ± 0.02	5.28 ± 0.06	*<0.0001*
**PI**	107.10 ± 1.11	117.60 ± 0.68	*<0.0001*

The values are given as mean ± standard error (*n* = 12)

*∑SFA* Sum of saturated fatty acids, *∑MUFA* Sum of monounsaturated fatty acids, *∑PUFA* Sum of polyunsaturated fatty acids, *∑n3-PUFA* Sum of polyunsaturated omega-3 fatty acids, *∑n6-PUFA* Sum of omega-6 polyunsaturated fatty acids, *DHA* Docosahexaenoic acid, *EPA* Eicosapentaenoic acid, *IA* Atherogenic index, *IT* Thrombogenic index, *h/H* *ratio* (hypocholesterolemic/hypercholesterolemic ratio), *PI* Peroxidation index

The nutritionally important indices, including the atherogenic (AtheroI), thrombogenic (TI) and hypocholesterolemic/hypercholesterolemic (h/H) ratios, as well as the peroxidation index (PI), are shown in Table 10. The best nutritional indices (AI, TI and h/H ratio) were observed in the PBM group, while the PI was significantly higher than in the FM group.

The results of the estimated indices of lipid metabolism are shown in Table 11. Considering FM and PBM, there were significant differences between the two dietary groups for thioesterase, ∆9 desaturase (C18), and **∆** 9 desaturase (C16 + C18), which were significantly higher in the PBM group than in the FM group, and for the estimated activities of elongase and **∆**9 desaturase (C16), which were significantly lower in the PBM group than in the FM group. On the other hand, the estimated activities of the enzymes ∆5 + ∆6 desaturase (n6) and ∆5 + ∆6 desaturase n3 were similar in the FM and PBM groups.

**Table 11:** Estimated indices of lipid metabolism in rainbow trout fillets fed with the two experimental diets

	FM	PBM	*p*-value
**Thioesterase**	6.38 ± 0.56	17.12 ± 0.91	*<0.001*
**Elongase**	0.24 ± 0.01	0.23 ± 0.01	*<0.05*
**∆9 desaturase (C16)**	16.78 ± 1.55	5.9 ± 0.53	*<0.001*
**∆9 desaturase (C18)**	91.15 ± 0.58	92.89 ± 0.45	*<0.0001*
**∆9 desaturase (C16 + C18)**	68.28 ± 0.96	71.20 ± 0.92	*<0.0001*
**∆5 + ∆6 desaturase n6**	5.24 ± 0.47	5.32 ± 0.62	0.742
**∆5 + ∆6 desaturase n3**	85.83 ± 1.19	85.17 ± 0.85	0.177

Values are presented as mean ± standard error (*n *= 12)

C12:0 = lauric acid; C14:0 = myristic acid; C14:1 = myristoleic acid; C15:0 = pentadecanoic acid; C16:0 = palmitic acid; C16:1 = palmitoleic acid; C17:0 = heptadecanoic acid; C17:1 = heptadecenoic acid; C18:0 = stearic acid; C18:1n9 = oleic acid; C18:1n7 = cis-vaccenoic acid; C18:2n6 = linoleic acid; C18:3n6 = γ-linolenic acid; C18:3n3 = *α*-linolenic acid; C20:0 = arachidic acid; C20:1n9 = eicosaenoic acid; C20:2n6 = eicosadienoic acid; C20:3n6 = dihomo-γ-linolenic acid; C20:4n3 = eicosatetraenoic acid; C20:4n6 = arachidonic acid; C20:5n3 = eicosapentaenoic acid; C22:0 = behenic acid; C22:1n9 = erucic acid; C22:5n3 = docosapentaenoic acid; C22:6n3 = docosaenoic acid; C24:1n9 = nervonic acid.

### FEEDNETICS™ validation for trial results

**Fig. 4:**
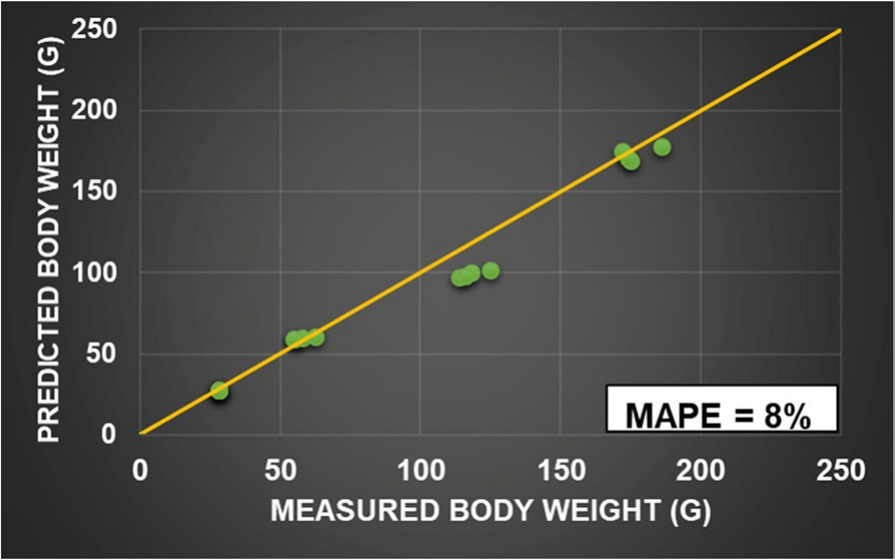
Comparison between the predicted and observed values and the mean absolute percentage error (MAPE)

The validation results showed that the FEEDNETICS™ effectively predicted the growth pattern of rainbow trout fed the experimental diet over a 90-day period with a MAPE of 8% (Fig. 4). The model deviation is likely due to incomplete input data, particularly the exclusion of digestibility data, as well as fatty acid composition, which are key factors influencing fish growth and nutrient utilization, respectively.

**Fig. 5:**
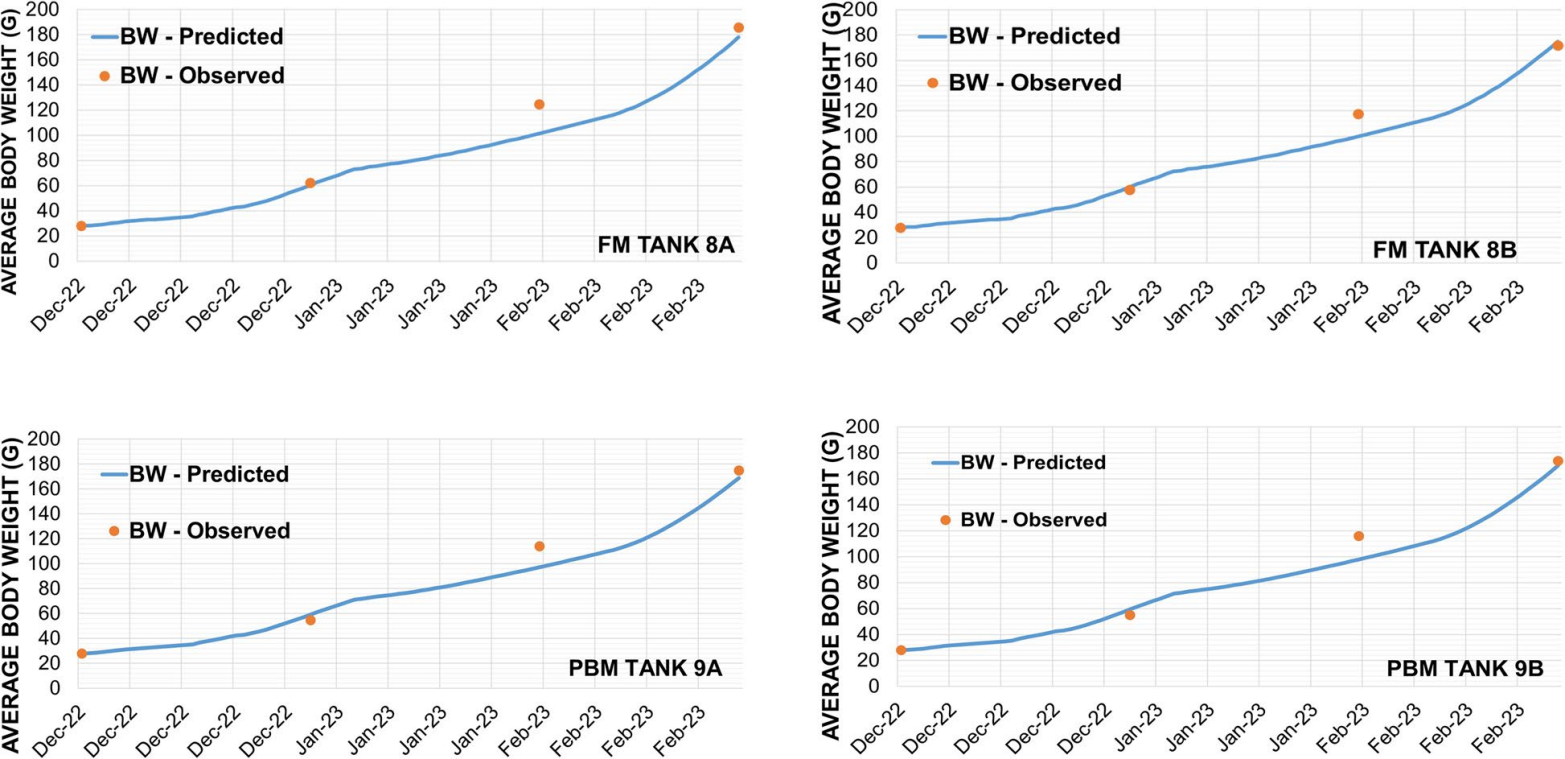
Results of model validation for experimental conditions. The graphs show the time series of the predicted and observed average body weight of fish growth over the experimental period, with the lines representing the fish growth predicted by the model and the red dots representing the average fish weight observed in the experiment

The comparison between the predicted and observed growth of rainbow trout fed different experimental diets over the trial period is presented in Fig. 5. A comparison was made between the data from the two experimental feeds and four fish tanks (FM: tanks 8 A, 8 B and PBM: tanks 9 A, 9 B). The FEEDNETICS™ model, while overall accurate in predicting growth trends, exhibited a consistent underestimation of fish body weights across all tanks. Specifically, the predicted weights for the FM and PBM groups were slightly higher than the actual weights recorded at the end of the trial period. The observed discrepancies may arise from several factors including incomplete input data used in the model.

The comparison of the results from the last sampling period and the predictions are summarized in Table 12. The results are presented at the end of the forecast period. According to the model, fish fed the FM diet showed better growth and feed utilization performance compared to fish fed the PBM diet. The predictions show that the average body weight of the FM-fed fish would reach 195.81 g, while the PBM-fed fish would weigh 184.45 g on average. In addition, the model predicts a lower FCR of 1.14 for the FM group, while the PBM group had a higher FCR of 1.23, indicating differences in feed efficiency between the two groups of fish.

**Table 12:** Validation scenario of the performance of rainbow trout fed with experimental diets using FEEDNETICS™

Indicator	FM	PBM
Average body weight (g/fish)	195.81	184.45
Growth rate - cumulative (%BW/day)	2.18	2.12
FCR (g feed/g BW gain)	1.14	1.23
Economic conversion ratio (€ feed/kg biomass gain)	1.37	1.47
Total N waste (kg N/ton biomass gain)	52.18	57.98
Total P waste (kg P/ton biomass gain)	5.85	6.28

However, when the economic performance, represented by the economic conversion rate (ECR) and the water nutrient waste indicators such as total nitrogen (N) and phosphorus (P) were analyzed, the result changed. From an economic point of view, the FM diet proved to be the more cost-effective option with lower feeding costs compared to PBM diets. According to the predictions of the FEEDNETICS™ model, FM was associated with better performance metrics compared to PBM.

Specifically, FM was predicted to lead to a 0.09-unit improvement in FCR. In addition, FM was expected to lead to a reduction in total N and P wastes by approximately − 5.8 and − 0.43 units, respectively, compared to PBM. These results suggest that FM might offer advantages over PBM in aquaculture in terms of feed efficiency and waste reduction.

## Discussion

Research interest in non-ruminant PAPs has surged, particularly since their recent reintroduction into the EU aquafeed industry. Among these, PBM is widely recognized for its nutritional value, comparable to that of FM. The use of PBM in aquaculture has shown varying degrees of success across different species [63]. Numerous studies have looked at the effects of PBM, but the results of these studies are inconclusive. The results of the present study show that the replacement of FM with PBM effectively fulfils the growth requirements of rainbow trout. This result aligns with numerous studies indicating that replacing FM with PBM in a ratio of 25–50% is optimal for most carnivorous fish [32]. A meta-analysis summarizing the effectiveness of PBM in aquaculture feed formulations used data from 47 published articles covering 33 different fish species [32]. The analysis revealed that many freshwater fish species can tolerate up to 100% FM replacement with PBM, while most fish species can accept up to 50% PBM in their diet without any issues [32].

As [64] indicates, the composition and digestibility of feather meal can vary significantly. These fluctuations can be attributed to differences in processing methods and the quality of raw materials. To achieve a more balanced amino acid profile in the diet, FMH should be combined with other protein meals. This combination helps to ensure that the nutritional requirements of the fish are met more effectively, promoting optimal growth and health. At the end of the experiment, all fish weighed six times their initial weight, regardless of the feed they received, and survival rates were consistent across all groups. However, there were observable trends in growth metrics between PBM-fed and FM-fed fish. Specifically, the PBM-fed fish exhibited a slightly lower WG and SGR compared to the FM-fed fish. Additionally, there was a trend toward a higher FCR in the PBM-fed fish, indicating potentially less efficient feed utilization in this group. It is important to note that these differences, while indicative of a trend, were not statistically significant according to the analysis performed. This suggests that, while PBM-fed fish may have demonstrated a slight decrease in growth performance, the extent of this decrease was not enough to be considered statistically different from the FM-fed group. This observation aligns with previous research findings. For instance, in studies with sea bass, replacing 50% of FM with 20% or 25% PBM resulted in growth comparable to that of fish fed a high FM control diet [65]. Shapawi et al. [66] also reported no significant differences in growth for juvenile humpback grouper fed diets containing 50% or 75% PBM protein. However, they did observe reduced growth in juvenile fish fed a diet with 100% PBM protein.

Our previous research on rainbow trout showed that trout fed diets high in PBM (55–70%) exhibited growth rates comparable to those of fish fed a control diet rich in FM at 37.3% without PBM [28]. Similar findings have been reported for marine fish species such as sea bream (*Sparus aurata*) and red sea bream (*P. major*), where complete replacement of FM with PBM did not negatively affect growth parameters or overall productivity, highlighting the adaptability of these fish to alternative protein sources [23, 67].

Knowledge of the effects of PBM on fish morphology and potential health consequences remains limited. The health of the digestive system, including the liver, is crucial for feed digestion and nutrient absorption, significantly affecting nutrient utilization [68]. No significant changes were observed in the gut and liver of the experimental groups, suggesting that PBM is a safe alternative feed ingredient in aquaculture. These findings are consistent with those of [65], who reported no significant histopathological changes in the liver or intestine of European seabass fed diets containing up to 25% PBM.

In our current study, histological analysis of the liver revealed no significant differences in lipid accumulation between the two feeding groups. To our knowledge, there is limited information on the histological analysis of the livers of rainbow trout fed PBM. In [69], the replacement of FM with PBM in the diet of Nile tilapia (*Oreochromis niloticus*) resulted in no detectable effects on the histological examination of liver tissue up to 100% replacement. Similar to our results, the livers showed normally shaped hepatocytes. Conversely [70], reported significant lipid accumulation in the liver and histological changes indicative of early steatosis in seabass fed an FM-free diet in which 40% plant protein was replaced with insects or PBM. The combination of insect meal and PBM partially mitigated these effects. In a study by [42], enlarged hepatocytes and liver steatosis were observed in Japanese seabass fed a mixture of 40% PBM, 35% meat and bone meal, 20% spray-dried blood meal, and 5% hydrolyzed feather meal, with hepatocyte vacuoles resembling lipid accumulation.

Furthermore, the inclusion of PBM in the diet had no negative effects on the morphometric indices of the foregut or hindgut, with the exception of villus height in the AI and submucosa width in the PI. PBM has an effect on the anatomical features or dimensions of the digestive tract structures, demonstrating compatibility with the digestive physiology of the organism. However, there were statistically significant effects on villus height in the AI of PBM-fed fish, which had greater villus height compared to FM-fed fish. In addition, the width of the submucosa in the PI of PBM-fed fish was significantly thicker and larger than that of FM-fed fish due to the infiltration of inflammatory cells in the submucosal layer. Despite these changes, the overall histological and morphometric characteristics of the intestinal mucosa were similar between the experimental groups, indicating that PBM was well tolerated by the trout. In line with these results, our recent study [65] showed that replacing part of the FM with insect meal and PBM had no negative effects on the proximal or distal gut of European seabass. This substitution significantly improved all morphometric parameters of the intestine and reduced both the degree of vacuolization and the extent of cellular infiltration.

In this study, higher levels of SCFAs, particularly butyrate and acetic acid, were found in the feces of trout fed PBM. This finding is consistent with previous studies in which sea bass fed exuviae or PBM meal also showed increased concentrations of acetate and butyrate in their feces compared to trout fed FM [63]. Similarly, trout fed pupal exuviae meal had the highest concentrations of SCFAs, especially butyrate, in their feces [71]. These SCFAs are the main products of bacterial fermentation of chitin produced by chitinolytic bacteria such as *Paenibacillus*, which were also found in greater abundance in gilthead seabream, in the insect meal-fed group [72]. SCFAs, including butyrate, play a crucial role in fish gut health, barrier function and mucosal immunity. These compounds have an anti-inflammatory effect, which is supported by studies showing that they can promote intestinal health in fish [73–76]. Butyric acid, an important cross-talk molecule, is known to have several beneficial effects on fish gut health and immunity [77, 78].

The substitution of FO with VeraMaris^®^ algae oil resulted in a significant alteration in the fatty acid composition of the fish fillets. Specifically, we observed a decrease in the levels of certain PUFAs traditionally found in marine-derived fish oils, while other beneficial fatty acids were enhanced. This change in fatty acid profile has implications for both fish health and the nutritional value of the fish for human consumption. For instance, while the reduction in some omega-3 fatty acids might affect fish growth and immune function, the inclusion of algae oil introduces a more sustainable source of these essential nutrients. This result aligns with previous findings that highlight the impact of dietary lipid sources on the fatty acid profile in fish fillets.

Our analysis of the fatty acids in the PBM diet revealed differences in the levels of PUFAs compared to the FM diet. The PBM diet had a lower content of n-3 PUFAs, particularly eicosapentaenoic acid (EPA, C20:5n3), while the docosahexaenoic acid (DHA, C22:6n3) levels were not significantly different between the two diets. Additionally, the PBM diet contained higher levels of n-6 PUFAs, including linoleic acid (C18:2n6). These dietary changes were mirrored in the fatty acid profile of the fish fillets, demonstrating a notable influence of the diet on the muscle fatty acid composition after 90 days of feeding Conversely, saturated fatty acids such as myristic acid (C14:0), palmitic acid (C16:0), monounsaturated fatty acids such as C16:1, cis-vaccenic acid (C18:1n7), eicosapentaenoic acid (EPA C20:5n3), and docosapentaenoic acid (DPA C22:5n3) decreased significantly due to the increased PBM and reduced FM and FO content in the diet.

In general, the ratio of n-3/n-6 PUFA in the diet increases the more FM is replaced by PBM, as PBM tends to be deficient in n-3 PUFA [79, 80]. Indeed, trout fed a diet in which only 20% FM was replaced with PBM showed increased n-3 and n-6 PUFA levels in their muscles, which is consistent with results in Nile tilapia [69] and juvenile barramundi [80] fed a diet based on 100% PBM protein. The substitution resulted in a reduction in EPA and DHA, typically abundant in marine-derived FO. However, VeraMaris^®^ algae oil provided an alternative source of omega-3s, which can help partially offset the reduction in EPA and DHA. Despite this shift, it is important to note that algae oil contributes to a more sustainable source of essential fatty acids, which is beneficial for the long-term viability of aquaculture practices.

Studies in humans have linked increased plasma cholesterol levels and an increased risk of cardiovascular disease with the intake of myristic acid (C14:0) intake [81]. Therefore, reducing myristic acid in the diet could benefit human health. In our study, the PBM-based diet contained lower amounts of myristic acid than the FM-based control diet. For the fish, a diet low in marine-derived EPA and DHA could affect growth performance, immune function, and overall physiological health. Omega-3 fatty acids are crucial for maintaining cell membrane fluidity, modulating inflammatory responses, and supporting proper growth.

For human consumers, the altered fatty acid profile presents a different nutritional composition. While the reduction in EPA and DHA might be considered a drawback from a nutritional standpoint, VeraMaris^®^ algae oil does provide a more balanced omega-3 to omega-6 ratio. This balance is important for cardiovascular health and has been linked to various health benefits, including a reduced risk of chronic diseases.

The content of omega-3 fatty acids in trout muscle exceeded 0.6 g/100 g, which is considered the standard value for this species [82]. The fillets of fish fed PBM contained more total omega-3 (such as EPA and DHA) than those of the FM group. This is likely due to the inclusion of algae oil in the PBM diet, which is also rich in omega-3. Although there was an increase in total omega-3 fatty acids, this did not result in a significant difference in the Σn3/Σn6 FA ratio index, in contrast to the results of [80]. In contrast, in another study, replacing FM with *Hermetia illucens* larval meal resulted in a consistent increase in omega-6 and a decrease in omega-3 fatty acids in fish muscle [83], as well as a decrease in the Σn3/Σn6 FA ratio and PUFA/SFA ratio [84]. The substitution of FO with VeraMaris^®^ algae oil also has significant implications from a sustainability perspective.

Fish is considered a valuable food for the prevention of coronary heart disease in humans, as it has a high content of PUFAs. Lower AtheroI and TI values indicate a lower risk of heart disease. Conversely, a higher h/H ratio is considered beneficial [80]. In our study, PBM diet had lower AI and TI values and a higher h/H ratio than FM diet, probably due to a higher PUFA content, as also observed by [83, 85, 86] in similar studies. This suggests that consumption of fish fed a balanced PBM diet could improve the nutritional value of rainbow trout fillet and provide similar health benefits compared to fish fed FM-based diets.

Animals can convert EFAs to LC-PUFAs depending on the activity level of elongases and desaturases, especially Δ5 and Δ6; moreover, the activity level of these enzymes depends on the LC-PUFA richness of the animal’s dietary substrate. In fact, the conversion of alpha-linolenic acid (18:3n-3) to eicosapentaenoic acid (EPA 20:5n-3) and then to docosahexaenoic acid (DHA 22:6n-3) occurs in many species of freshwater fish and is significantly reduced in marine fish due to the abundance of EPA and DHA in the marine environment. In contrast, the dietary substrate of freshwater fish is rich in EFA (linoleic acid and alpha-linolenic acid) and, to a lesser extent, EPA, but not DHA. Consequently, despite their common origin, freshwater fish differ significantly from marine fish in terms of essential fatty acid requirements. This reflects the different distribution of fatty acids in the two environments and the consequent different evolutionary pressures, such as the different need to possess enzymes that can complete the conversion of EFAs to LC-PUFAs [87].

It seems noteworthy to comment on the Δ5-Δ6-desaturase activity performed on the n3 and n6 series. The differences in the fatty acid profile between the fillets of the FM and PBM groups suggest that the enzymes involved in the desaturation of long-chain and very long-chain fatty acids were modulated by the inclusion of algae in the diet [88]. The acidic composition of fillets differed significantly between groups; fish fed algae oil (PBM group) had the highest levels of n3 and n6 PUFA and EPA + DHA, even though Δ5-Δ6 desaturase activity, both n3 and n6 series, was similar. Recent studies have investigated the possibility that algae ingested in the diet increase the levels of n3-PUFA in fish muscle [89–91], due to their fatty acid composition [92]. The results of the lipid profile confirmed this hypothesis and encouraged the researchers to conduct further studies on the effects of algae on lipid deposition in rainbow trout.

The FEEDNETICS™ application model used to analyze the fish growth data in our experiment provided valuable insights for validation. Impressively, the FEEDNETICS™ model accurately predicted body weight for this experimental dataset and had a MAPE of around 8%. This emphasizes the reliability of the model in evaluating feeding strategies for trout on commercial farms. However, closer inspection revealed some intriguing discrepancies between the simulation results generated by FEEDNETICS™ and the actual observations recorded during the experiment. Interestingly, although the FEEDNETICS™ simulation results showed a slightly higher average body weight for the FM group than for the PBM group. Indeed, the FEEDNETICS™ simulation results showed that the average body weight of the FM group was 195.81 g, which was slightly higher than that of the PBM group (with an average body weight of 184.45 g). However, in the experimental trial, the final body weight was higher in the FM group (170.80 g) than in the PBM group (166.91 g). These differences suggest that although the model provided a good prediction overall, certain factors – such as incomplete input data – may have influenced its accuracy.

Similarly, the FEEDNETICS™ simulation yielded an FCR of 1.14 in the FM group and 1.23 in the PBM group, indicating differences in feed efficiency between the two trout groups. In contrast, the experimental trial yielded an FCR of 1.33 in the FM group and 1.37 in the PBM group, in which the FM was replaced by a combination of PM, FMH and omega-3 oil based on natural VeraMaris^®^ algae oil. Thus, these FCR values obtained from the actual trial did not match the FCR results predicted by the FEEDNETICS™ simulation. It was expected that FM diet would improve FCR by 0.09 units and reduce total N and P in wastewater by approximately − 5.8 and − 0.43 units, respectively, compared to PBM. This suggests that FM could provide better feed efficiency and waste reduction in aquaculture.

No significant differences were found between the experimental test and the FEEDNETICS™ simulation in terms of growth. FEEDNETICS™ predicted that fish fed the FM diet would grow slightly better than those fed the PBM- fed fish, but the difference was not significant. The differences between the FEEDNETICS™ simulation data and the actual experimental data can be explained by some factors and limitations. One important factor is that the FEEDNETICS™ model did not have data on digestibility, which provides information on how efficiently the fish utilize the nutrients from their feed. This data was not available and was therefore not included in the model. In this scenario, the validation process relied on the default data provided by the model, which is likely to reflect generalized assumptions about the growth and nutrient utilization of rainbow trout. The performance of the model can be improved by incorporating more accurate and comprehensive input data specific to rainbow trout strains and populations.

## Conclusions

Our study demonstrated that replacing up to 20% of FM in the diet of rainbow trout with a combination of PBM and FMH, along with fully substituting FO with VeraMaris^®^ natural algae oil, does not compromise growth performance, feed conversion, gut morphology or liver health. Although there was a reduction in PUFAs in the fillets, the nutritional quality indices of fish muscle improved in PBM-fed fish. This included lower AtheroI and TI indices, as well as a higher h/H ratio, which suggests potential health benefits for human consumers, such as a reduced risk of cardiovascular diseases. These findings indicate that PBM and natural algal oil are promising and viable alternatives to FM and FO in trout nutrition, enhancing the sustainability and potential health benefits of trout production. Furthermore, the use of FEEDNETICS™ supports environmentally friendly and economically viable practices in fish farming by identifying sustainable feed ingredients like PBM and natural algal oil. It also aids in developing feeding strategies that optimize growth performance while minimizing environmental impact.

## Data Availability

Data is provided within the manuscript or supplementary information files.

## Abbreviations

AI: Anterior intestine
AtheroI: Atherogenicity index
AOAC: Association of Official Analytical Chemists
BP: Biomass production
BW: Body weight
CF: Condition factor
DHA: Docosasaenoic acid
EAA: Essential amino acids
ECR: Economic conversion ratio
EPA: Eicosapentaenoic acid
FA: Fatty acid
FBW: Final body weight
FCR: Feed Conversion Ratio
FM: Fishmeal
FMH: Feather meal hydrolysate
FO: Fish oil
h/H: Hypocholesterolemic/hypercholesterolemic
H_2_SO_4_: Sulphuric acid
He: Helium
IBW: Initial body weight
MAPE: Mean absolute percentage error
MT: Muscle thickness
MUFAS: Monounsaturated fatty acids
N: Nitrogen
NBF: Neutral buffered formalin
NEAA: Non-essential amino acids
P: Phosphorus
PAPs: Processed animal proteins
PBM: Poultry by-product meal
PI: Peroxidation index
PI: Posterior intestine
PUFAs: Polyunsaturated fatty acids
SCFAs: Short‑chain fatty acids
SEM: Standard error of the mean
SGR: Specific growth rate
SL: Standard-length
SR: Survival rate
TI: Thrombogenicity index
VIH: Villus height
VIH: Villus width
WG: Weight gain

## References

[1] Hardy RW. Utilization of plant proteins in fish diets: effects of global demand and supplies of fishmeal. Aquac Res. 2010;41(5):770–6. 10.1111/j.1365-2109.2009.02349.x.

[2] Péron G, François Mittaine J, Le Gallic B. Where do fishmeal and fish oil products come from? An analysis of the conversion ratios in the global fishmeal industry. Mar Policy. 2010;34(4):815–20. 10.1016/j.marpol.2010.01.027.

[3] Lazzarotto V, Corraze G, Leprevost A, Quillet E, Dupont-Nivet M, Médale F. Three-year breeding cycle of rainbow trout (*Oncorhynchus mykiss*) fed a plant-based diet, totally free of marine resources: consequences for reproduction, fatty acid composition and progeny survival. PLoS ONE. 2015;10(2):e0117609. 10.1371/journal.pone.0117609. 25658483 10.1371/journal.pone.0117609 PMC4320095

[4] Ytrestøyl T, Aas TS, Åsgård T. Utilisation of feed resources in production of Atlantic salmon (*Salmo salar*) in Norway. Aquaculture. 2015;448:365–74. 10.1016/j.aquaculture.2015.06.023.

[5] Naylor RL, Goldburg RJ, Primavera JH, Kautsky N, Beveridge MCM, Clay J, Folke C, Lubchenco J, Mooney H, Troell M. Effect of aquaculture on world fish supplies. Nature. 2000;405(6790):1017–24. 10.1038/35016500. 10890435 10.1038/35016500

[6] Gatlin IIIDM, Barrows FT, Brown P, Dabrowski K, Gaylord TG, Hardy RW, Herman E, Hu G, Krogdahl Å, Nelson R, et al. Expanding the utilization of sustainable plant products in aquafeeds: a review. Aquac Res. 2007;38(6):551–79. 10.1111/j.1365-2109.2007.01704.x.

[7] Colombo SM. Physiological considerations in shifting carnivorous fishes to plant-based diets. Fish Physiol. 2020;38:53–82. 10.1016/bs.fp.2020.09.002.

[8] Aragão C, Gonçalves AT, Costas B, Azeredo R, Xavier MJ, Engrola S. Alternative proteins for fish diets: implications beyond growth. Animals. 2022;12(9):1211. 10.3390/ani12091211. 35565636 10.3390/ani12091211 PMC9103129

[9] Cardinaletti G, Di Marco P, Daniso E, Messina M, Donadelli V, Finoia MG, Petochi T, Fava F, Faccenda F, Contò M, et al. Growth and welfare of rainbow trout (*Oncorhynchus mykiss*) in response to graded levels of insect and poultry by-product meals in fishmeal-free diets. Animals. 2022;12(13):1698. 10.3390/ani12131698. 35804596 10.3390/ani12131698 PMC9264821

[10] Davis DA, Arnold CR. Replacement of fish meal in practical diets for the pacific white shrimp, *Litopenaeus vannamei*. Aquaculture. 2000;185(3–4):291–8. 10.1016/S0044-8486(99)00354-3.

[11] Hernández C, Olvera-Novoa M, Hardy RW, Hermosillo A, Reyes C, Gonzalez B. Complete replacement of fish meal by porcine and poultry by-product meals in practical diets for fingerling Nile tilapia Oreochromis niloticus: Digestibility and growth performance. Aquac Nutr. 2009;16;44–53. 10.1111/j.1365-2095.2008.00639.x.

[12] Hegedüs M, Bokori J, Andrásofszky E, Kövári L. Optimizing protein quality of mixtures of blood meal, feather meal and bone meal. Acta Vet Hung. 1990;38(3):143–52. 2099600

[13] Fasakin EA, Serwata RD, Davies SJ. Comparative utilization of rendered animal derived products with or without composite mixture of soybean meal in hybrid tilapia (*Oreochromis niloticus×Oreochromis mossambicus*) diets. Aquaculture. 2005;249(1–4):329–38. 10.1016/j.aquaculture.2005.02.059.

[14] Yu HR, Zhang Q, Cao H, Wang XZ, Huang GQ, Zhang BR, Fan JJ, Liu SW, Li WZ, Cui Y. Apparent digestibility coefficients of selected feed ingredients for juvenile snakehead, *Ophiocephalus Argus*. Aquac Nutr. 2013;19(2):139–47. 10.1111/j.1365-2095.2012.00947.x.

[15] González-Rodríguez Á, Celada JD, Carral JM, Sáez-Royuela M, García V, Fuertes JB. Evaluation of poultry by-product meal as partial replacement of fish meal in practical diets for juvenile tench (*Tinca Tinca L*). Aquac Res. 2016;47(5):1612–21. 10.1111/are.12622.

[16] Campos I, Matos E, Marques A, Valente LMP. Hydrolyzed feather meal as a partial fishmeal replacement in diets for European seabass (*Dicentrarchus labrax*) juveniles. Aquaculture. 2017;476:152–9. 10.1016/j.aquaculture.2017.04.024.

[17] Yu Y. Replacement of fish meal with poultry by-product meal and hydrolyzed feather meal in feeds for finfish. In: "Alternative Protein Sources in Aquaculture Diets". Boca Raton: CRC Press; 2008. ISBN 978-1-00-342121-4.

[18] Zho QC, Zhao J, Li P, Wang HL, Wang LG. Evaluation of poultry by-product meal in commercial diets for juvenile cobia (*Rachycentron canadum*). Aquaculture. 2011;322–323:122–7. 10.1016/j.aquaculture.2011.09.042.

[19] Psofakis P, Karapanagiotidis IT, Malandrakis EE, Golomazou E, Exadactylos A, Mente E. Effect of fishmeal replacement by hydrolyzed feather meal on growth performance, proximate composition, digestive enzyme activity, haematological parameters and growth-related gene expression of gilthead seabream (*Sparus aurata*). Aquaculture. 2020;521:735006. 10.1016/j.aquaculture.2020.735006.

[20] Karapanagiotidis IT, Psofakis P, Mente E, Malandrakis E, Golomazou E. Effect of fishmeal replacement by poultry by-product meal on growth performance, proximate composition, digestive enzyme activity, haematological parameters and gene expression of gilthead seabream (*Sparus aurata*). Aquac Nutr. 2019;25(1):3–14. 10.1111/anu.12824.

[21] Fontinha F, Magalhães R, Moutinho S, Santos R, Campos P, Serra CR, Aires T, Oliva-Teles A, Peres H. Effect of dietary poultry meal and oil on growth, digestive capacity, and gut microbiota of Gilthead Seabream (*Sparus aurata*) juveniles. Aquaculture. 2021;530:735879. 10.1016/j.aquaculture.2020.735879.

[22] Psofakis P, Meziti A, Berillis P, Mente E, Kormas KA, Karapanagiotidis IT. Effects of dietary fishmeal replacement by poultry by-product meal and hydrolyzed feather meal on liver and intestinal histomorphology and on intestinal microbiota of Gilthead Seabream (*Sparus aurata*). Appl Sci. 2021;11(19): 8806. 10.3390/app11198806.

[23] Dawson MR, Alam MS, Watanabe WO, Carroll PM, Seaton PJ. Evaluation of poultry by-product meal as an alternative to fish meal in the diet of juvenile black sea bass reared in a recirculating aquaculture system. North Am J Aquac. 2018;80(1):74–87. 10.1002/naaq.10009.

[24] Takakuwa F, Murashita K, Noguchi Y, Inui T, Watanabe K, Sugiyama S, Yamada S, Biswas A, Tanaka H. Effects of long-term feeding of fishmeal-free diet on growth parameters, bile acid status, and bile acid-related gene expression of yearling red sea bream *pagrus major* (Temminck & Schlegel, 1843). Aquaculture. 2023;570: 739444. 10.1016/j.aquaculture.2023.739444.

[25] Burr GS, Wolters WR, Barrows FT, Hardy RW. Replacing fishmeal with blends of alternative proteins on growth performance of rainbow trout (*Oncorhynchus mykiss*), and early or late stage juvenile atlantic salmon (*Salmo salar*). Aquaculture. 2012;334–337:110–6. 10.1016/j.aquaculture.2011.12.044.

[26] Hatlen B, Jakobsen JV, Crampton V, Alm M, Langmyhr E, Espe M, Hevrøy EM, Torstensen BE, Liland N, Waagbø R. Growth, feed utilization and endocrine responses in atlantic salmon (*Salmo salar*) fed diets added poultry by-product meal and blood meal in combination with poultry oil. Aquac Nutr. 2015;21(5):714–25. 10.1111/anu.12194.

[27] Barreto-Curiel F, Parés-Sierra G, Correa-Reyes G, Durazo-Beltrán E, Viana MT. Total and partial fishmeal substitution by poultry by-product meal (Petfood grade) and enrichment with acid fish silage in aquafeeds for juveniles of rainbow trout *Oncorhynchus mykiss*. Lat Am J Aquat Res. 2016;44(2):327–35. 10.3856/vol44-issue2-fulltext-13.

[28] Rimoldi S, Terova G, Ascione C, Giannico R, Brambilla F. Next generation sequencing for gut microbiome characterization in rainbow trout (*Oncorhynchus mykiss*) fed animal by-product meals as an alternative to fishmeal protein sources. PLoS ONE. 2018;13(3): e0193652. 10.1371/journal.pone.0193652. 29509788 10.1371/journal.pone.0193652 PMC5839548

[29] Randazzo B, Zarantoniello M, Gioacchini G, Cardinaletti G, Belloni A, Giorgini E, Faccenda F, Cerri R, Tibaldi E, Olivotto I. Physiological response of rainbow trout (*Oncorhynchus mykiss*) to graded levels of *Hermetia illucens* or poultry by-product meals as single or combined substitute ingredients to dietary plant proteins. Aquaculture. 2021;538: 736550. 10.1016/j.aquaculture.2021.736550.

[30] Maiolo S, Parisi G, Biondi N, Lunelli F, Tibaldi E, Pastres R. Fishmeal partial substitution within Aquafeed formulations: Life Cycle Assessment of Four Alternative protein sources. Int J Life Cycle Assess. 2020;25:1455–71. 10.1007/s11367-020-01759-z.

[31] Maiolo S, Cristiano S, Gonella F, Pastres R. Ecological sustainability of aquafeed: an emergy assessment of novel or underexploited ingredients. J Clean Prod. 2021;294: 126266. 10.1016/j.jclepro.2021.126266.

[32] Galkanda-Arachchige HSC, Wilson AE, Davis DA. Success of fishmeal replacement through poultry by-product meal in aquaculture feed formulations: a meta-analysis. Rev Aquac. 2020;12(3):1624–36. 10.1111/raq.12401.

[33] Yu H, Li M, Yu L, Ma X, Wang S, Yuan Z, Li L. Partial replacement of fishmeal with poultry by-product meal in diets for coho salmon (*Oncorhynchus kisutc*h) post-smolts. Animals. 2023;13(17): 2789. 10.3390/ani13172789. 37685053 10.3390/ani13172789 PMC10487097

[34] Franks B, Ewell C, Jacquet J. Animal welfare risks of global aquaculture. Sci Adv. 2021;7(14):eabg0677. 10.1126/sciadv.abg0677. 33811081 10.1126/sciadv.abg0677 PMC11057778

[35] Vis JW van de, Kolarevic J, Stien LH, Kristiansen TS, Gerritzen MA, Braak K van de, Abbink W, Saether BS, Noble C. Welfare of farmed fish in different production systems and operations. In: The welfare of fish. Cham: Springer; 2020. p. 323–361.

[36] Glencross B, Baily J, Berntssen M, Hardy R, Mackenzie S, Tocher D. Risk assessment of the use of alternative animal and plant raw material resources in aquaculture feeds. Rev Aquac. 2019;12(2):703–58. 10.1111/raq.12347.

[37] Ciji A, Akhtar MS. Stress management in aquaculture: a review of dietary interventions. Rev Aquac. 2021;13(4):2190–247. 10.1111/raq.12565.

[38] Martínez-Llorens S, Baeza-Ariño R, Nogales-Mérida S, Jover-Cerdá M, Tomás-Vidal A. Carob seed germ meal as a partial substitute in gilthead sea bream (*Sparus aurata*) diets: amino acid retention, digestibility, gut and liver histology. Aquaculture. 2012;338–341:124–33. 10.1016/j.aquaculture.2012.01.029.

[39] Ye H, Zhou Y, Su N, Wang A, Tan X, Sun Z, Zou C, Liu Q, Ye C. Effects of replacing fish meal with rendered animal protein blend on growth performance, hepatic steatosis and immune status in hybrid grouper (*Epinephelus fuscoguttatus♀ × Epinephelus lanceolatus♂*). Aquaculture. 2019;511: 734203. 10.1016/j.aquaculture.2019.734203.

[40] Zhou Z, Yao W, Ye B, Wu X, Li X, Dong Y. Effects of replacing fishmeal protein with poultry by-product meal protein and soybean meal protein on growth, feed intake, feed utilization, gut and liver histology of hybrid grouper (*Epinephelus fuscoguttatus ♀ × Epinephelus lanceolatus ♂*) juveniles. Aquaculture. 2020;516: 734503. 10.1016/j.aquaculture.2019.734503.

[41] Chaklader MR, Siddik MAB, Fotedar R. Total replacement of fishmeal with poultry by-product meal affected the growth, muscle quality, histological structure, antioxidant capacity and immune response of juvenile barramundi, *lates calcarifer*. PLoS ONE. 2020;15(11): e0242079. 10.1371/journal.pone.0242079. 33180835 10.1371/journal.pone.0242079 PMC7661056

[42] Hu L, Yun B, Xue M, Wang J, Wu X, Zheng Y, Han F. Effects of fish meal quality and fish meal substitution by animal protein blend on growth performance, flesh quality and liver histology of Japanese seabass (*Lateolabrax japonicus*). Aquaculture. 2013;372–5. 10.1016/j.aquaculture.2012.10.025.

[43] Føre M, Frank K, Norton T, Svendsen E, Alfredsen JA, Dempster T, Eguiraun H, Watson W, Stahl A, Sunde LM, et al. Precision fish farming: a new framework to improve production in aquaculture. Biosyst Eng. 2018;173:176–93. 10.1016/j.biosystemseng.2017.10.014.

[44] Zhou C, Xu D, Lin K, Sun C, Yang X. Intelligent feeding control methods in aquaculture with an emphasis on fish: a review. Rev Aquac. 2018;10(4):975–93. 10.1111/raq.12218.

[45] Lugert V, Thaller G, Teten J, Schulz C, Krieter J. A review on fish growth calculation: multiple functions in fish production and their specific application. Rev Aquac. 2016;8(1):30–42. 10.1111/raq.12071.

[46] Chary K, Brigolin D, Callier MD. Farm-scale models in fish aquaculture – an overview of methods and applications. Rev Aquac. 2022;14(4):2122–57. 10.1111/raq.12695.

[47] Soares FMRC, Nobre AMD, Raposo AIG, Mendes RCP, Engrola SAD, Rema PJAP, Conceição LEC, Silva TS. Development and application of a mechanistic nutrient-based model for precision fish farming. J Mar Sci Eng. 2023;11(3):472. 10.3390/jmse11030472.

[48] Nutrient requirements of fish and shrimp; National Academies Press: Washington, D.C., 2011. ISBN 978-0-309-16338-5.

[49] AOAC. Official methods of analysis. 21st ed. Gaithersburg, MD, USA: Association of Official Analytical Chemists International; 2019.

[50] Oteri M, Di Rosa AR, Lo Presti V, Giarratana F, Toscano G, Chiofalo B. Black soldier fly larvae meal as alternative to fish meal for aquaculture feed. Sustainability. 2021;13(10):5447. 10.3390/su13105447.

[51] Caballero MJ, Izquierdo MS, Kjørsvik E, Fernández AJ, Rosenlund G. Histological alterations in the liver of sea bream, Sparus Aurata L., caused by short- or long-term feeding with vegetable oils. Recovery of normal morphology after feeding Fish Oil as the Sole lipid source. J Fish Dis. 2004;27(9):531–41. 10.1111/j.1365-2761.2004.00572.x. 15357712 10.1111/j.1365-2761.2004.00572.x

[52] Rodrigues S, Antunes SC, Nunes B, Correia AT. Histological alterations in gills and liver of rainbow trout (*Oncorhynchus mykiss*) after exposure to the antibiotic oxytetracycline. Environ Toxicol Pharmacol. 2017;53:164–76. 10.1016/j.etap.2017.05.012. 28599186 10.1016/j.etap.2017.05.012

[53] Ruiz A, Andree KB, Sanahuja I, Holhorea PG, Calduch-Giner JÀ, Morais S, Pastor JJ, Pérez-Sánchez J, Gisbert E. Bile salt dietary supplementation promotes growth and reduces body adiposity in gilthead seabream (*Sparus Aurata*). Aquaculture. 2023;566:739203. 10.1016/j.aquaculture.2022.739203.

[54] Escaffre AM, Kaushik S, Mambrini M. Morphometric evaluation of changes in the digestive tract of rainbow trout (*Oncorhynchus mykiss*) due to fish meal replacement with soy protein concentrate. Aquaculture. 2007;273(1):127–38. 10.1016/j.aquaculture.2007.09.028.

[55] Chlebicz-Wójcik A, Śliżewska K. The effect of recently developed synbiotic preparations on dominant fecal microbiota and organic acids concentrations in feces of piglets from nursing to fattening. Animals. 2020;10(11): 1999. 10.3390/ani10111999. 33143237 10.3390/ani10111999 PMC7693995

[56] Folch J, Lees M, Sloane Stanley GH. A simple method for the isolation and purification of total lipides from animal tissues. J Biol Chem. 1957;226(1):497–509. 10.1016/S0021-9258(18)64849-5. 13428781

[57] Christie WW. Preparation of Ester Derivatives of Fatty Acids for Chromatographic Analysis. Adv Lipid Methodol. 1993;2:69–111.

[58] Ulbricht TL, Southgate DA. Coronary heart disease: seven dietary factors. Lancet (London England). 1991;338:985–92. 10.1016/0140-6736(91)91846-m. 1681350 10.1016/0140-6736(91)91846-m

[59] Santos-Silva J, Bessa RJB, Santos-Silva FJLPS. Effect of genotype, feeding system and slaughter weight on the quality of light lambs: II. Fatty acid composition of meat. Livest Prod Sci. 2002;77(2–3):187–94. 10.1016/S0301-6226(02)00059-3.

[60] Luciano G, Pauselli M, Servili M, Mourvaki E, Serra A, Monahan FJ, Lanza M, Priolo A, Zinnai A, Mele M. Dietary olive cake reduces the oxidation of lipids, including cholesterol, in lamb meat enriched in polyunsaturated fatty acids. Meat Sci. 2013;93(3):703–14. 23273482 10.1016/j.meatsci.2012.11.033

[61] Mattioli S, Duarte JMM, Castellini C, D’Amato R, Regni L, Proietti P, Businelli D, Cotozzolo E, Rodrigues M, Dal Bosco A. Use of olive leaves (whether or not fortified with sodium selenate) in rabbit feeding: Effect on performance, carcass and meat characteristics, and estimated indexes of fatty acid metabolism. Meat Sci. 2018;143:230–6. 10.1016/j.meatsci.2018.05.010. 29803133 10.1016/j.meatsci.2018.05.010

[62] Hammer Øyvind, Harper DAT Ryan. Past: Paleontological Statistics Software Package for Education and Data Analysis. Palaeontol Elect. 2001;4(1):9.

[63] Cardinaletti G, Di Marco P, Daniso E, Messina M, Donadelli V, Finoia MG, Petochi T, Fava F, Faccenda F, Contò M, Cerri R, Volpatti D, Bulfon C, Mandich A, Longobardi A, Marino G, Fernanda L, Rodriguez P, Parisi G. Growth and welfare of rainbow trout (*Oncorhynchus mykiss*) in response to graded levels of insect and poultry by-product meals in fishmeal-free diets. Animals. 2022;12(13): 1698. 10.3390/ani12131698. 35804596 10.3390/ani12131698 PMC9264821

[64] Pfeuti G, Longstaffe J, Brown LS, Shoveller AK, Taylor CM, Bureau DP. Disulphide bonds and cross-linked amino acids may affect amino acid utilization in feather meal fed to rainbow trout (*Oncorhynchus mykiss*). Aquac Res. 2019;50(8):2081–95. 10.1111/are.14079.

[65] Rimoldi S, Di Rosa AR, Armone R, Chiofalo B, Hasan I, Saroglia M, Kalemi V, Terova G. The replacement of fish meal with poultry by-product meal and insect exuviae: effects on growth performance, gut health and microbiota of the European seabass, *Dicentrarchus labrax*. Microorganisms. 2024;12(4):744. 10.3390/microorganisms12040744. 38674688 10.3390/microorganisms12040744 PMC11052083

[66] Shapawi R, Ng WK, Mustafa S. Replacement of fish meal with poultry by-product meal in diets formulated for the humpback grouper, *Cromileptes altivelis*. Aquaculture. 2007;273(1):118–26. 10.1016/j.aquaculture.2007.09.014.

[67] Sabbagh M, Schiavone R, Brizzi G, Sicuro B, Zilli L, Vilella S. Poultry by-product meal as an alternative to fish meal in the juvenile gilthead seabream (*Sparus aurata*) diet. Aquaculture. 2019;511: 734220. 10.1016/j.aquaculture.2019.734220.

[68] Caimi C, Gasco L, Biasato I, Malfatto V, Varello K, Prearo M, Pastorino P, Bona MC, Francese DR, Schiavone A, et al. Could dietary black soldier fly meal inclusion affect the liver and intestinal histological traits and the oxidative stress biomarkers of siberian sturgeon (Acipenser Baerii) juveniles? Animals. 2020;10(1):155. 10.3390/ani10010155. 31963360 10.3390/ani10010155 PMC7022867

[69] Aydin B, Gümüş E, Balci BA. Effect of dietary fish meal replacement by poultry by-product meal on muscle fatty acid composition and liver histology of fry of nile tilapia, *Oreochromis niloticus* (Actinopterygii: Perciformes: Cichlidae). Acta Ichthyol Piscat. 2015;45(4):343–51. 10.3750/AIP2015.45.4.02.

[70] Donadelli V, Di Marco P, Mandich A, Finoia MG, Cardinaletti G, Petochi T, Longobardi A, Tibaldi E, Marino G. Effects of dietary plant protein replacement with insect and poultry by-product meals on the liver health and serum metabolites of sea bream (*Sparus Aurata*) and sea bass (*Dicentrarchus labrax*). Animals. 2024;14(2):241. 10.3390/ani14020241. 38254412 10.3390/ani14020241 PMC10812684

[71] Rimoldi S, Ceccotti C, Brambilla F, Faccenda F, Antonini M, Terova G. Potential of shrimp waste meal and insect exuviae as sustainable sources of chitin for fish feeds. Aquaculture. 2023;567:739256. 10.1016/j.aquaculture.2023.739256.

[72] Rimoldi S, Di Rosa AR, Oteri M, Chiofalo B, Hasan I, Saroglia M, Terova G. The impact of diets containing hermetia illucens meal on the growth, intestinal health, and microbiota of gilthead seabream (*Sparus aurata*). Fish Physiol Biochem. 2024;1–22. 10.1007/s10695-024-01314-9. 10.1007/s10695-024-01314-9 PMC11213805 38386264

[73] Canani RB, Costanzo MD, Leone L, Pedata M, Meli R, Calignano A. Potential beneficial effects of butyrate in intestinal and extraintestinal diseases. World J Gastroenterol. 2011;17(12):1519–28. 10.3748/wjg.v17.i12.1519. 21472114 10.3748/wjg.v17.i12.1519 PMC3070119

[74] Terova G, Díaz N, Rimoldi S, Ceccotti C, Gliozheni E, Piferrer F. Effects of sodium butyrate treatment on histone modifications and the expression of genes related to epigenetic regulatory mechanisms and immune response in European sea bass (*Dicentrarchus labrax*) fed a plant-based diet. PLoS ONE. 2016;11(7): e0160332. 10.1371/journal.pone.0160332. 27471849 10.1371/journal.pone.0160332 PMC4966935

[75] Tran NT, Li Z, Wang S, Zheng H, Aweya JJ, Wen X, Li S. Progress and perspectives of short-chain fatty acids in aquaculture. Rev Aquac. 2020;12:283–98. 10.1111/raq.12317.

[76] Tran NT, Liang H, Li J, Deng T, Zhang M, Li S. Health benefits of butyrate and its producing bacterium, *Clostridium butyricum*, on aquatic animals. Fish Shellfish Immunol Rep. 2023;4: 100088. 10.1016/j.fsirep.2023.100088. 36910329 10.1016/j.fsirep.2023.100088 PMC9995936

[77] Abdel-Latif HMR, Abdel-Tawwab M, Dawood MAO, Menanteau-Ledouble S, El-Matbouli M. Benefits of dietary butyric acid, sodium butyrate, and their protected forms in aquafeeds: a review. Rev Fish Sci Aquac. 2020;28(4):421–48. 10.1080/23308249.2020.1758899.

[78] Tawfick MM, Xie H, Zhao C, Shao P, Farag MA. Inulin fructans in diet: role in gut homeostasis, immunity, health outcomes and potential therapeutics. Int J Biol Macromol. 2022;208:948–61. 10.1016/j.ijbiomac.2022.03.218. 35381290 10.1016/j.ijbiomac.2022.03.218

[79] Norambuena F, Hermon K, Skrzypczyk V, Emery JA, Sharon Y, Beard A, Turchini GM. Algae in fish feed: performances and fatty acid metabolism in juvenile atlantic salmon. PLoS ONE. 2015;10(4): e0124042. 10.1371/journal.pone.0124042. 25875839 10.1371/journal.pone.0124042 PMC4398455

[80] Siddik MAB, Chungu P, Fotedar R, Howieson J. Bioprocessed poultry by-product meals on growth, gut health and fatty acid synthesis of juvenile barramundi, *Lates Calcarifer* (Bloch). PLoS ONE. 2019;14(4):e0215025. 10.1371/journal.pone.0215025. 30964913 10.1371/journal.pone.0215025 PMC6456252

[81] Briggs MA, Petersen KS, Kris-Etherton PM. Saturated fatty acids and cardiovascular disease: replacements for saturated fat to reduce cardiovascular risk. Healthcare. 2017;5(2):29. 10.3390/healthcare5020029. 28635680 10.3390/healthcare5020029 PMC5492032

[82] Estévez A, Frade P, Ferreira M, Regueiro L, Alvarez M, Blanco B, Fernández L, Soula M. Effects of alternative and sustainable ingredients on rainbow trout (*Oncorhynchus mykiss*) growth, muscle composition and health. Aquac J. 2022;2(2):37–50. 10.3390/aquacj2020004.

[83] Renna M, Schiavone A, Gai F, Dabbou S, Lussiana C, Malfatto V, Prearo M, Capucchio MT, Biasato I, Biasibetti E, et al. Evaluation of the suitability of a partially defatted black soldier fly (*Hermetia illucens L*) Larvae meal as ingredient for rainbow trout (*Oncorhynchus mykiss Walbaum*) diets. J Anim Sci Biotechnol. 2017;8:1–13. 10.1186/s40104-017-0191-3. 28680591 10.1186/s40104-017-0191-3 PMC5494141

[84] Józefiak A, Nogales-Mérida S, Mikołajczak Z, Rawski M, Kierończyk B, Mazurkiewicz J. The utilization of full-fat insect meal in rainbow trout () nutrition: the effects on growth performance, intestinal microbiota and gastrointestinal tract histomorphology. Ann Anim Sci. 2019;19(3):747–65. 10.2478/aoas-2019-0020.

[85] Belforti M, Gai F, Lussiana C, Renna M, Malfatto V, Rotolo L, De Marco M, Dabbou S, Schiavone A, Zoccarato I, et al. Tenebrio molitor meal in rainbow trout (*Oncorhynchus mykiss*) diets: effects on animal performance, nutrient digestibility and chemical composition of fillets. Ital J Anim Sci. 2015;14(4):4170. 10.4081/ijas.2015.4170.

[86] Terova G, Moroni F, Antonini M, Bertacchi S, Pesciaroli C, Branduardi P, Labra M, Porro D, Ceccotti C, Rimoldi S. Using glycerol to produce European sea bass feed with oleaginous microbial biomass: effects on growth performance, filet fatty acid profile, and FADS2 gene expression. Front Mar Sci. 2021;8: 715078. 10.3389/fmars.2021.715078.

[87] Tocher DR. Metabolism and functions of lipids and fatty acids in Teleost Fish. Rev Fish Sci. 2003;11(2):107–84. 10.1080/713610925.

[88] Peixoto MJ, Salas-Leitón E, Pereira LF, Queiroz A, Magalhães F, Pereira R, Abreu H, Reis PA, Gonçalves JFM, Ozório RODA. Role of dietary seaweed supplementation on growth performance, digestive capacity and immune and stress responsiveness in European seabass (*Dicentrarchus labrax*). Aquacult Rep. 2016;3:189–97. 10.1016/j.aqrep.2016.03.005.

[89] Dantagnan P, Hernandez A, Borquez A, Mansilla A. Inclusion of macroalgae meal (*Macrocystis pyrifera*) as feed ingredient for rainbow trout (*Oncorhynchus mykiss*): effect on flesh fatty acid composition. Aquac Res. 2009;41(1):87–94. 10.1111/j.1365-2109.2009.02308.x.

[90] Guroy B, Ergun S, Merrifield DL, Guroy D. Effect of autoclaved Ulva meal on growth performance, nutrient utilization and fatty acid profile of rainbow trout, *Oncorhynchus mykiss*. Aquacult Int. 2013;21:605–15. 10.1007/s10499-012-9592-7.

[91] Wilke T, Faulkner S, Murphy L, Kealy L, Kraan S, Brouns F. Seaweed enrichment of feed supplied to farm-raised Atlantic salmon (*Salmo salar*) is associated with higher total fatty acid and LC n-3 PUFA concentrations in fish flesh. Eur J Lipid Sci Technol. 2015;117(6):767–72. 10.1002/ejlt.201400166.

[92] Holdt SL, Kraan S. Bioactive compounds in seaweed: functional food applications and legislation. J Appl Phycol. 2011;23:543–97. 10.1007/s10811-010-9632-5.

